# Elucidating the genetic basis of antioxidant status in lettuce (*Lactuca sativa*)

**DOI:** 10.1038/hortres.2015.55

**Published:** 2015-11-25

**Authors:** Annabelle Damerum, Stacey L Selmes, Gaia F Biggi, Graham JJ Clarkson, Steve D Rothwell, Maria José Truco, Richard W Michelmore, Robert D Hancock, Connie Shellcock, Mark A Chapman, Gail Taylor

**Affiliations:** 1 Centre for Biological Sciences, University of Southampton, Life Sciences, University Road, Southampton SO17 1BJ, UK; 2 Vitacress Limited, Lower Link Farm, St Mary Bourne, Andover, Hampshire SP11 6DB, UK; 3 The Genome Centre and the Department of Plant Sciences, University of California, Davis, CA 95616, USA; 4 The James Hutton Institute, Invergowrie, Dundee DD2 5DA, UK

## Abstract

A diet rich in phytonutrients from fruit and vegetables has been acknowledged to afford protection against a range of human diseases, but many of the most popular vegetables are low in phytonutrients. Wild relatives of crops may contain allelic variation for genes determining the concentrations of these beneficial phytonutrients, and therefore understanding the genetic basis of this variation is important for breeding efforts to enhance nutritional quality. In this study, lettuce recombinant inbred lines, generated from a cross between wild and cultivated lettuce (*Lactuca serriola* and *Lactuca sativa*, respectively), were analysed for antioxidant (AO) potential and important phytonutrients including carotenoids, chlorophyll and phenolic compounds. When grown in two environments, 96 quantitative trait loci (QTL) were identified for these nutritional traits: 4 for AO potential, 2 for carotenoid content, 3 for total chlorophyll content and 87 for individual phenolic compounds (two per compound on average). Most often, the *L. serriola* alleles conferred an increase in total AOs and metabolites. Candidate genes underlying these QTL were identified by BLASTn searches; in several cases, these had functions suggesting involvement in phytonutrient biosynthetic pathways. Analysis of a QTL on linkage group 3, which accounted for >30% of the variation in AO potential, revealed several candidate genes encoding multiple MYB transcription factors which regulate flavonoid biosynthesis and flavanone 3-hydroxylase, an enzyme involved in the biosynthesis of the flavonoids quercetin and kaempferol, which are known to have powerful AO activity. Follow-up quantitative RT-PCR of these candidates revealed that 5 out of 10 genes investigated were significantly differentially expressed between the wild and cultivated parents, providing further evidence of their potential involvement in determining the contrasting phenotypes. These results offer exciting opportunities to improve the nutritional content and health benefits of lettuce through marker-assisted breeding.

## Introduction

Dietary consumption of plant phytochemicals from fruits and vegetables has been linked to positive health effects^1–5^ Antioxidants (AOs) may contribute to this benefit since they can scavenge free radicals, such as singlet oxygen and superoxide radicals, potentially reducing oxidative damage to cellular components,^6,7^ although this role remains controversial and is not universally accepted.^8^ These and other phytochemicals also have important roles in plants in defence against pests, pathogens, and UV light, attraction of pollinators and competitive interactions with other plants (reviewed in ref. 9). The largest group of phytonutrients is the phenolic compounds, including phenolic acids and flavonoids (anthocyanins, anthocyanidins, flavones, flavonols, flavanones, proanthocyanins and isoflavones^5^) and these have been shown to possess powerful AO activity *in vitro*.^10–13^ Carotenoids; yellow, orange and red terpenoids, are another group of plant compounds with AO activity, acting as accessory pigments during photosynthesis to quench the excited state of chlorophyll, and also to provide colouration.^14,15^ Many carotenoids have pro-vitamin A activity due to the presence of vitamin A as part of their structure, making them an important nutrient in the human diet, reducing the risk of respiratory diseases and blindness.^16^ The value of the photosynthetic pigment chlorophyll as an important phytochemical in foods has been underappreciated. Chlorophyll derivatives extracted from spinach have recently been shown to prevent DNA damage of human lymphocytes *in vitro* in a dose-dependent manner,^17^ suggesting they are key contributors to the overall AO potential of foods.

Although lettuce is not usually acknowledged as being a rich source of beneficial phytochemicals, it does contain phenolic compounds, vitamins C and E, and carotenoids.^18,19^ Lettuce consumption can improve cholesterol metabolism in rats and can stimulate the AO capacity of blood plasma in both humans and rats.^20,21^ Beneficial phenolic compounds in lettuce include chicoric acid (also called dicaffeoyltartaric acid), chlorogenic acid (also known as caffeoyl quinic acid) and the flavonoid quercetin.^18,22,23^ Chicoric acid extracted from lettuce inhibits both lipid peroxidation and cyclo-oxygenase enzyme activities; ^23^chlorogenic acid is effective at inhibiting the hypermethylation of DNA, which is characteristic of tumour cells.^24^ Finally, quercetin has potential anti-cancer properties, arresting A549 lung cancer cell lines *in vitro*.^25^ Despite the effects of these phenolics in isolation, it has been suggested that eating whole foods rich in natural sources of these beneficial compounds is more effective than relying solely on dietary supplements.^26–28^ Thus there is increasing pressure to develop new and novel germplasm with enhanced nutritional quality and to enable breeding programmes to use molecular markers effectively, with a clear understanding of the chemistry underlying nutritional traits.

Extensive DNA polymorphism data are available for lettuce. More than 35 000 lettuce genes have been analysed for single feature polymorphisms (SFPs) using a high-density Affymetrix GeneChip microarray^29^ and SFPs have been mapped to create an ultra-dense, gene-based, genetic linkage map for lettuce using a recombinant inbred line (RIL) population generated from a cross between *Lactuca sativa* (cultivated lettuce) and *L. serriola* (wild ‘prickly’ lettuce; http://chiplett.ucdavis.edu/).^30^ This resource has been useful in determining the genetic basis for traits such as disease resistance and shelf-life in lettuce.^31–33^We therefore used this extensively characterized mapping population to investigate the genetic basis of AO potential. The aim of this study was to identify quantitative trait loci (QTL) determining AO potential, total carotenoid, chlorophyll and phenol content, and levels of individual metabolites, as well as to identify candidate genes underlying these QTL. This provides underpinning information to develop molecular tools for breeding lettuce with enhanced nutritional qualities. Previously, QTL have been identified for AO capacity in tomato fruits,^34^ anthocyanin in raspberries,^35^ carotenoid content in maize,^36^ and chlorophyll and AO potential in lettuce,^37^ but to our knowledge, none have linked these traits to underlying metabolic signatures and candidate genes.

## Materials and methods

### Plant material

#### RIL mapping population

Sixty F_9_ RILs, generated from a cross between cultivated lettuce (Lactuca sativa cv. Salinas) and wild lettuce (*L. serriola* accession UC96US23), along with representatives of the parental lines, were used as the mapping population in this study. The RILs investigated are a subset of a total population of 213 RILs developed and characterized by the Compositae Genome Project (http://chiplett.ucdavis.edu/), which were determined to be highly informative during previous investigations.^32^ Five *L. sativa* cultivars, denoted C1-C5, including two red types (C1; Daredevil and C2; SSC 3025) and three green (C3; Frontrunner, C4; Thriller, C5; Carlsbad) were obtained from Shamrock Seeds Company (UK).

### Plant growth

#### Glasshouse experiments

Nine replicates of each of the RILs and parental lines were planted in a fully randomised blocked design (1–3 replicates per block and three blocks), with positions randomly selected using Minitab 14.0 (Minitab Inc., Philadelphia, PA, USA). Plants were grown in 70 × 70 × 80 mm pots containing blended peat, seed and modular growing media, at pH 5.5 (Vapogro, Kekkilä and Avoncrop Ltd, Windsor, UK). Initially, four seeds were sown per pot and thinned following germination so that only one plant per pot remained to grow to maturity. Day temperatures ranged from 18 °C to 27 °C and night temperatures averaged 18 °C, with day length approximately 16 h. Pots were watered from below when required. Following 5 weeks growth, whole plants at the rosette stage were harvested and leaves were ground to a fine powder in liquid N_2_ before storage at –80 °C.

#### Field experiment

Lettuce seeds were planted in September 2009 within a commercial crop of Lollo Rosso lettuce on a farm in Azenha do Mar, Odemira, Portugal (37°47′28″N, 8°79′18″W). Nine biological replicates were planted across a fully randomised and blocked design (see Glasshouse experiments), spaced at 0.1 m intervals in 1.2 m × 35.0 m beds and each block was surrounded with two rows of Cos lettuce to minimise edge effects.^32^ Plants were irrigated from above when required and all other conditions were as those for the surrounding commercial crop. Whole plants were harvested at the rosette stage after 5 weeks of growth and transported under refrigeration to the University of Southampton, UK, where leaves were snap frozen and stored at –80 °C.

### Determination of AO potential

The Ferric Reducing Antioxidant Power assay (FRAP assay; refs.^38,39^) was used to estimate the total AO potential of the RILs and RIL parents grown in the glasshouse and controlled environment experiments, according to the method revised by Payne *et al.*^39^ (See Supplementary Information for details).

### Extraction and quantification of chlorophyll and carotenoid content

Three 1 cm diameter discs were taken from the fourth true leaf of each plant of the glasshouse grown RILs at point of harvest, avoiding major veins; one from the tip and one from either side of the mid-rib vein. Pigments were extracted from leaf discs by incubating in microfuge tubes containing 500 μl of dimethylformamide in the dark at 4 °C for >48 h. Absorbance of the extracts was measured at 647, 664 and 480 nm in a cuvette spectrophotometer (U-2000, Hitachi, Wokingham, UK). Chlorophyll a, b, total chlorophyll and carotenoid concentration (all µg/ml) were calculated.^40^

### Determination of phenolic content

Phenols were extracted as outlined by Llorach *et al*.^19^ with modifications. Briefly, leaf material ground in liquid N_2_ was freeze-dried and 0.1–0.2 g of lyophilised leaf material was resuspended in 20 volumes of methanol:water:formic acid (25:24:3), vortexed rapidly and extracted in the dark at 4 °C for 30 min under continuous agitation. Samples were centrifuged (10 min, 13 000 rpm) and the supernatant was saved. The pellet was resuspended and re-extracted as described above, and the second supernatant was combined with the first. Extracts were kept in the dark at –20 °C until further analysis.

#### Total phenolic content

The enzymatic assay for total phenolic content was as outlined by Stevanto *et al*.,^41^ with modifications. A total of 100 μl of the above supernatant was diluted 10-fold with water and added to 900 μl of reaction buffer (0.1 M potassium phosphate buffer (pH 8.0), containing 20 mM hydrogen peroxide, 30 mM 4-aminophenazone and 100 U/ml horseradish peroxidase). Following a 5-min reaction period absorbance of each sample in triplicate was measured at 500 nm in spectrophotometric cuvettes. Aqueous solutions of catechin (0.1–1 mM), previously utilised as a standard for measuring phenolic content in lettuce,^18^ were utilised to generate a calibration curve from which total phenolic content of each sample was calculated as catechin equivalents, mg/ml dry weight (DW).

#### Identification and quantification of individual phenolics

A known concentration of the flavonol morin was added as an internal standard to the extracted phenols. *Liquid chromatography–mass spectrometry* (LC/MS)/MS was conducted on a Thermo HPLC system, consisting of an Acela autosampler and an Acela 600 pump (for further details see Supplementary Information). Compounds were identified using their UV absorption characteristics and parent and daughter ion masses as described.^19^ Relative quantification was achieved from the parent ion peak area, corrected according to the peak area of the morin internal standard.

### QTL analysis and identification of candidate genes

A dense linkage map of the RIL mapping population based on genic SFP markers was already available for QTL analysis (http://chiplett.ucdavis.edu/). A framework map consisting of 613 markers spaced approximately 3 cM apart across the 9 linkage groups (LGs) was used for the QTL analysis (Table 1; Supplementary Table S1). QTL mapping was conducted using composite interval mapping in Windows QTL Cartographer Ver. 2.5.^42^ Chromosome walk speed was set at 1 cM and the logarithm of odds (LOD) threshold for declaring a significant QTL (*P* < 0.05) was estimated for each trait by permutation tests with 1000 iterations.^43^ QTLs were plotted using MapChart 2.2.^44^ Co-localising QTLs were defined as two or more QTL with overlapping LOD intervals. Candidate genes within major QTL were identified in BLASTn searches based on their similarity to genes annotated and reported in the literature as having roles which could influence the levels of secondary metabolites (see Supplementary Information). Single nucleotide polymorphisms (SNPs) were identified in the predicted coding regions of genes under the LG3 AO QTL by aligning sequencing reads of *L. serriola* (UC96US23) and 4 of the RILs determined to have within the top 10 AO potential and 4 of the RILs in the bottom 10 lowest AO potential (reads for all of the highest and lowest ranking RILs for AO potential were not available) to the *L. sativa* cv. Salinas reference genome sequence and where sequencing reads were available, identifying SNP haplotypes. cDNA sequences of candidate genes for *L. sativa* cv. Salinas and *L. serriola* were downloaded via GenBank, translated using the ExPASy tool^45^ and aligned using Clustal^46^ to identify non-synonymous amino acid substitutions and deletions.

### Quantitative RT-PCR

Real-time qPCR was conducted to evaluate the expression of 10 candidate genes selected from the genomic regions underlying the LG3 AO QTL for the wild and cultivated parents of the mapping population. Three biological replicates of each parent from the field trial were analysed in duplicate for the candidates, along with two reference genes *ACT* and *40S* (see Supplementary Information for details).

### Statistical analysis

For the phenotype data, two sample *t*-testing and one-way analysis of variance (ANOVA) with post hoc Tukey’s testing were conducted on raw phenotype data using Minitab 16 (Minitab Ltd.) and mean data were evaluated via Pearson’s correlation coefficient analysis using SigmaPlot (Systat Software Inc.). Data were normalised by log-transformation when required. Differential expression between the wild and cultivated parents determined by quantitative RT-PCR was identified by two sample *t*-tests in R version 3.2.2.^47^

## Results

### Phenotyping the RIL mapping population

#### AO potential

**Figure 1:**
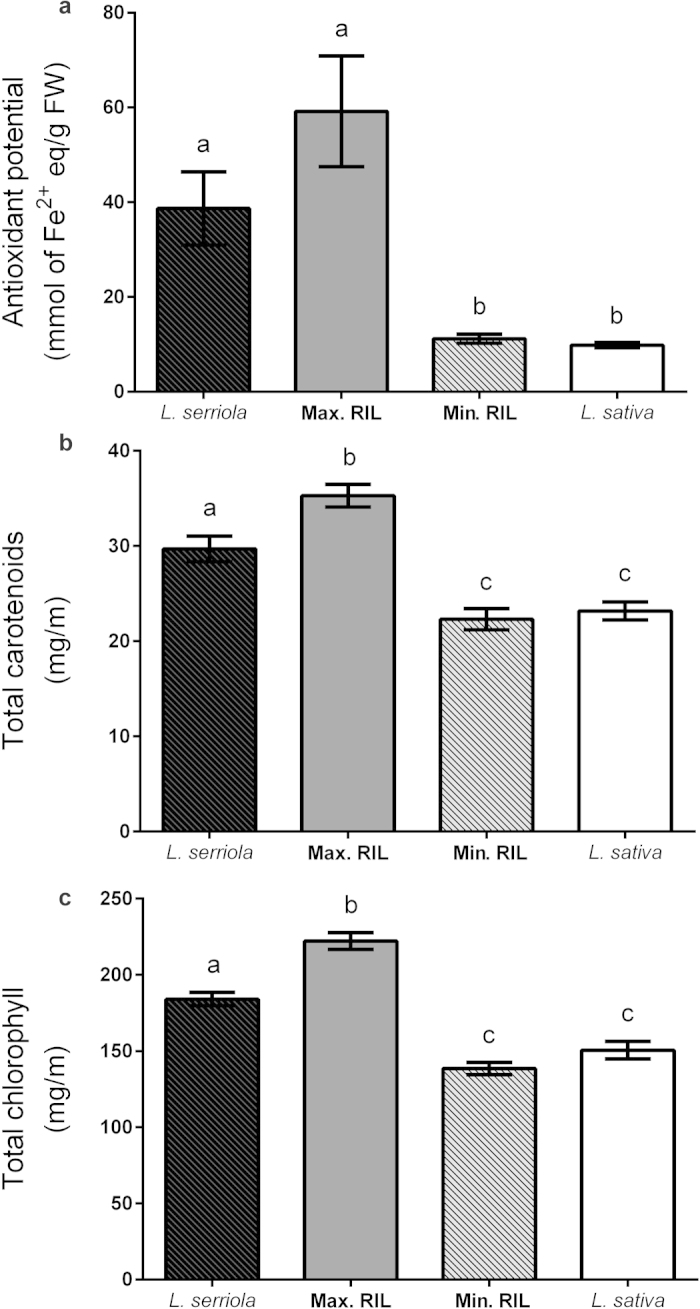
Phenotyping the RIL population. Analysis of antioxidant-related phenotypes in the parental lines (*L. sativa* and *L. serriola*), highest RIL (Max RIL) and lowest RIL (low Min RIL). Letters indicate significant differences. (**a**) total antioxidant potential (one-way ANOVA; *F*_3,32_ = 11.38, *P* ≤ 0.001), (**b**) total carotenoids (*F*_3,31_ = 27.09, *P* ≤ 0.001) and (**c**) total chlorophyll (*F*_3,31_ = 58.63, *P* ≤ 0.01). Bars represent the mean ± standard error.

The AO potential of *L. serriola* acc. UC96US23, the wild parent of the RILs, was over threefold greater than that of *L. sativa* cv. Salinas, the cultivated parent (38.68 ± 7.72 vs. 9.83 ± 0.53 mmol, respectively; one-way ANOVA, *F*_3,32_=11.38, *P* < 0.001). No significant differences were observed between the RIL with the highest AO potential (59.17 ± 11.69 mmol) and *L. serriola* nor the RIL with the lowest AO potential (11.19 ± 0.96 mmol) and *L. sativa*; however, there was evidence of transgressive segregation in that some RILs had a higher AO potential than the *L. serriola* parent (Figure 1A).

#### Total carotenoid and chlorophyll content

Both chlorophyll and carotenoid content were higher in the wild parent than in the cultivated one (one-way ANOVA, *F*_3,31_=58.63, *P* < 0.01 and *F*_3,31_=27.09, *P* < 0.001, respectively). Although carotenoid content was similar between *L. sativa* (20.52 ± 2.79 mg/m) and the lowest scoring RIL (22.30 ± 1.12 mg/m), the RIL scoring the greatest value for these traits (35.28 ± 1.20 mg/m) was significantly higher than *L. serriola* (29.69 ± 1.34 mg/m; Figure 1; *F*_3,31_ = 27.09, *P* < 0.001), again suggesting the presence of transgressive segregation. Similarly, chlorophyll content was significantly higher in the RIL with the highest value than *L. serriola* (22212 ± 16.69 vs. 184.07 ± 13.25 mg/m; Figure 1C; *F*_3,31_ = 58.63, *P* < 0.01).

#### Phenolic profile

**Figure 2:**
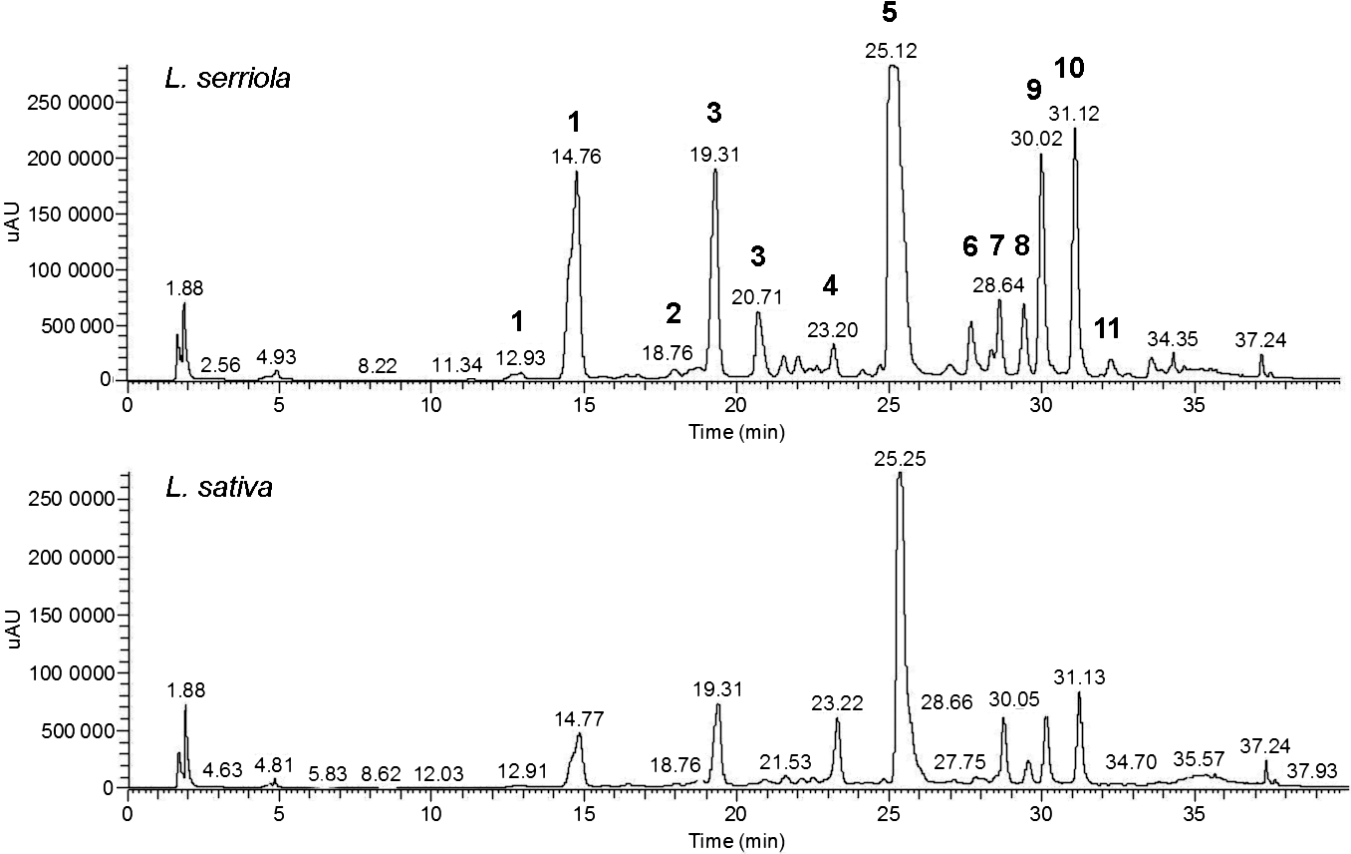
Comparison of the phenolic profiles of *L. serriola* and *L. sativa*. LC-MS profiles of the RIL parents, *Lactuca serriola* and *L. sativa*. Figure shows the relative maxima of absorbance of samples against retention time in (minutes), also displayed above each peak. Peaks: (1) CTA; (2) 5-CoQA (5-p-coumaroylquinic acid); (3) caffeoyl quinic acid; (4) caffeoyl malic acid; (5) di-CTA; (6) di-pCT; (7) dicaffeoyl quinic acid; (8) kaempferol glucuronide; (9) quercetin-3-glucuronide; (10) quercetin-3-malonylglucoside; (11) kaempferol-3-malonylglucoside. Multiple peaks for the same compound indicate isoforms.

Clear differences in total phenolic content were detected between wild and cultivated lettuce (38.76 ± 4.65 vs. 22.25 ± 1.25 catechin equivalent, mg/g dry weight, respectively; two-sample *t*-test, t_(8)_=–3.43, *P* ≤ 0.01). Individual phenolic compounds were further quantified by LC/MS/MS. Visual inspection of the LC-MS profiles revealed clear qualitative and quantitative differences in phenolic composition for the two parent lines (Figure 2). Metabolites such as caffeoyl tartaric acid (CTA), 5-p-coumaroylquinic acid (5-CoQA), caffeoyl quinic acid (CQA), di-pCT, kaempferol glucuronide (K-3Gc), quercetin-3-glucoronide (Q-3Gc), quercetin-3-malonylglucoside (Q-3MG) and kaempferol-3-malonylglucoside (K-3MG; peaks 1–3, 6 and 8–11, respectively, on Figure 2) were present at greater concentrations in *L. serriola* than *L. sativa*, whilst concentrations of di-CTA (DCTA; chicoric acid) and dicaffeoyl quinic acid (DCQA; peaks 5 and 7, respectively; Figure 2) were comparable and caffeoyl malic acid (peak 4; Figure 2) was the only phenolic compound noticeably present at greater abundance in *L. sativa*.

A total of 23 individual metabolites were detected from the leaf samples of the RILs grown in the glasshouse environment. These consisted of two CTA isoforms (CTA1 and CTA2), two di-caffeoyl tartaric acid isoforms (DCTA and mDCTA), caffeoyl malic acid (CMA), three caffeoyl quinic acid isoforms (CQA1, CQA2, CQA3), two di-caffeoylquinic acid isoforms (DCQA and 3,5-DCQA), two 5-coumaroylquinic acid isoforms (5-CoQA1 and 5-CoQA2), five quercetin derivatives (Q-3G, Q-3Gc, Q-3MG, Q-3MG-7Gc and Q-3MG-7G), two luteolin derivatives (L-7G and L-7Gc), two kaempferol derivatives (K-3Gc and K-3MG) and two unknown compounds, denoted 331 and 347 based on their m/z ratios (Supplementary Table S2). The above phenolics were also identified in the field grown RILs, excluding 5-CoQA2.

Relative quantities of each phenolic compound were estimated by comparison of the relative peak area to that of an internal standard. The top four most abundant metabolites were consistent amongst the field and glasshouse environments with chicoric acid (DCTA) found to be the most abundant in the RILs, comprising >30% of the total phenolic compounds in both the field and glasshouse grown RILs. Quercetin 3-malonylglucoside (Q-3MG) was identified as the second most abundant in both the field and glasshouse trials (18% and 16%, respectively). This was followed by CQA1 (11%) and Q-3Gc (10%) in the glasshouse grown RILs and Q-3Gc (10%) and CQA1 (9%) from the field.

**Figure 3:**
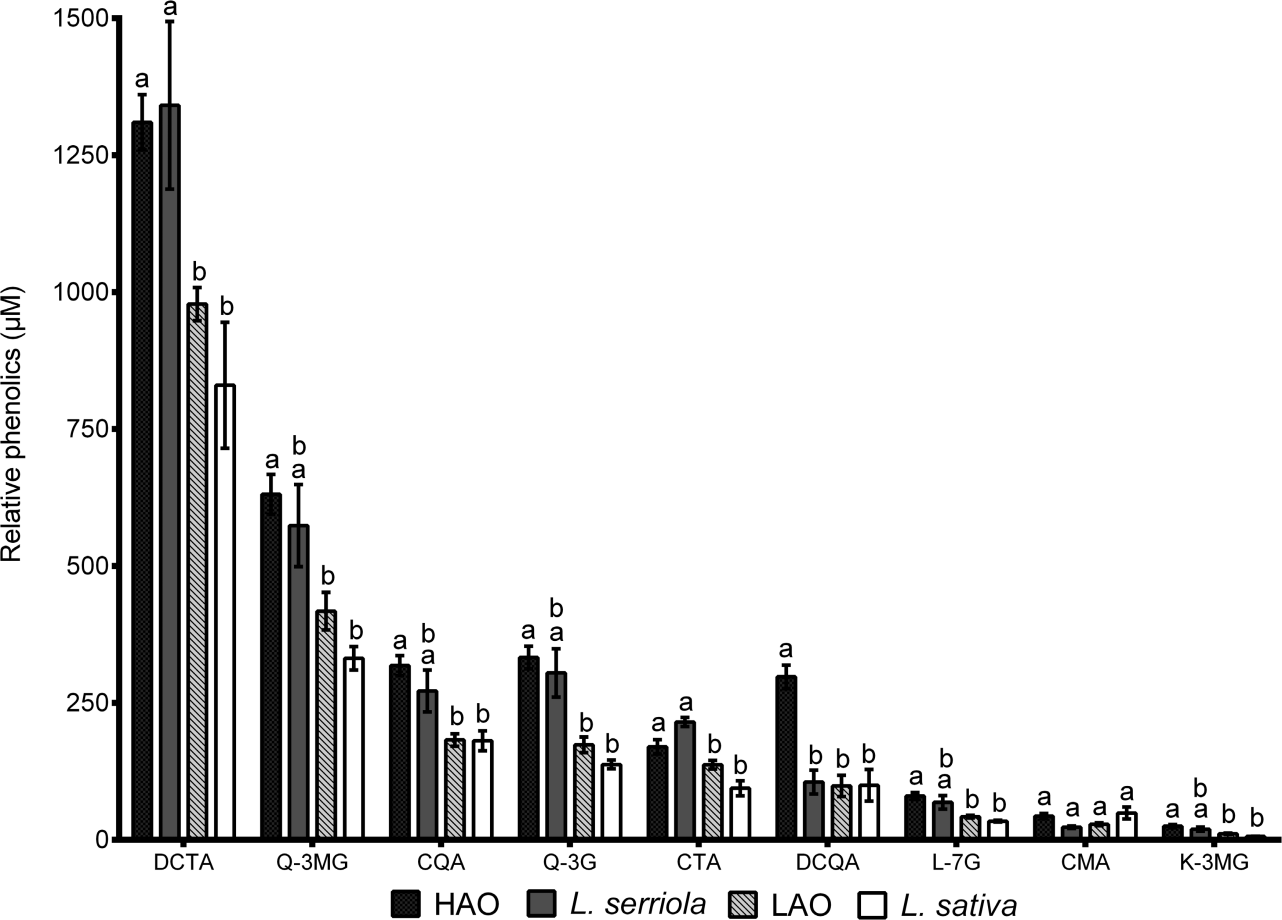
Comparison of relative phenolic concentrations amongst *L. serriola*, *L. sativa* and the high and low antioxidant RILs. Relative concentration of phenolics DCTA, Q-3MG, CQA, Q-3G, CTA, DCQA, L-7G, CMA and K-3MG for the parents and the four RILs measured to have the highest (HAO) and lowest (LAO) antioxidant status. Bars represent the mean ± standard error, with letters indicating significant differences (one-way ANOVA; see text for details).

Relative concentrations of the most abundant metabolites detected in lettuce were compared against *L. serriola* and *L. sativa* and for the top four RILs measured to have the highest (denoted HAO lines 1–4) and four RILs with the lowest (LAO lines 1–4) AO potential (Figure 3). DCTA concentration was significantly higher in the wild parent in comparison to the cultivated parent (one-way ANOVA, *F*_3,44_ = 26.26, *P* < 0.001) and this was also seen for CTA (*F*_3,44_ = 26.26, *P* < 0.001). Transgressive segregation of metabolites was often observed with concentrations usually higher in the HAO line than in the cultivated parent, seen for Q-3MG (*F*_3,44_ = 6.43, *P* < 0.01), CQA (*F*_3,44_ = 12.34, *P* < 0.001), Q-3G (*F*_3,44_ = 13.97, *P* < 0.001), CTA, DCQA (*F*_3,44_ = 17.39, *P* < 0.001), L-7G (*F*_3,44_=10.31, *P* < 0.01) and K-3MG (*F*_3,44_ = 6.33, *P* < 0.01), though there were no differences of relative CMA concentration amongst any of the lines (*F*_3,44_ = 2.27, *P *> 0.05). In most cases, the HAO line also had greater metabolite concentrations than the LAO, excluding for CMA. There were no differences in relative metabolite concentration between *L. sativa* and the LAO line excluding DCQA and no differences were observed between *L. serriola* and the HAO line.

#### AO potential is correlated with shelf life and various metabolites

Pearson’s correlation coefficient analysis of mean trait data revealed several significant correlations. AO potential was found to be positively correlated with shelf life (*P* < 0.05, Table 1), measured in the same RIL subset by Zhang *et al*.^32^ Total chlorophyll and carotenoids were strongly positively correlated with each other (*P* < 0.001) but negatively correlated with AO potential (*P* < 0.01). Relative abundances of Q-3MG-7Gc, K-3MG, the CQA isoforms 2 and 3, and 5-CoQA2 were all found to be positively correlated with AO potential measured for the glasshouse grown RILs. Shelf life was positively correlated with the flavonoids Q-3MG-7Gc and K-3MG (*P* < 0.05) and negatively correlated with total carotenoids (*P* < 0.05). AO potential was not found to significantly correlate with total phenolics and 5-CoQA2 was the only metabolite to significantly positively correlate with phenolic content (*P* < 0.01).

#### Relationship between AO potential and total phenolics

**Figure 4:**
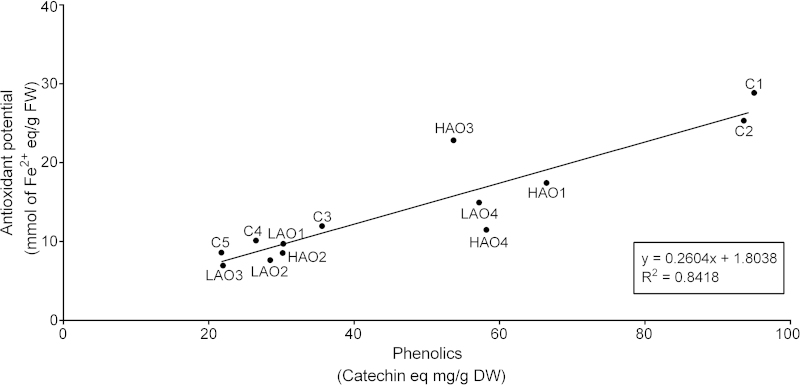
Relationship between antioxidant potential and total phenolics. Linear regression between antioxidant potential and total phenolics of the four RILs determined to have the highest AO potential (HAO 1–4) and four with the lowest AO potential (LAO 1–4), along with five *L. sativa* cultivars (C 1–5).

AO potential and total phenolics of the four RILs measured to have the highest antioxidant potential (HAO lines) and the four with the lowest (LAO lines) were reassessed in a subsequent trial alongside five commercial varieties. Generally, AO potential increased with total phenolics, with the HAO and LAO lines typically clustering (Figure 4). The two red cultivars C1 and C2 showed superior AO and phenolic status, but all other cultivars (C3–C5) clustered with the majority of the LAO lines (Figure 4). The interaction between AO potential and phenolic content was not as expected for HAO2 and LAO4, with HAO2 clustering with the LAO lines 1–3 and LAO4 with the other HAO lines (Figure 4).

### QTL analysis

A linkage map composed of 613 SFP markers distributed over the nine chromosomal LGs (http://chiplett.ucdavis.edu/) was utilised for QTL analysis. A total of 38 QTL from 24 traits were detected for the field trial and 62 QTL from 30 traits for the glasshouse trial, with QTL distributed across all nine LGs (Supplementary Table S5, Supplementary Figure S1).

#### AO potential

Four significant QTL were detected for AO potential measured in the glasshouse grown RILs, on LGs 3, 4, 7 and 9, accounting for 30%, 12%, 16% and 9% of the phenotypic variation (PV), respectively. For the QTL on LGs 3, 4 and 7 the alleles inherited from *L. serriola* resulted in an increase in the trait value whereas for the fourth QTL allele inheritance from *L. sativa* caused an increase in the trait.

#### Total carotenoid and chlorophyll content

Two QTL for total chlorophyll were detected on LGs 3 and 7, with allele inheritance from *L. sativa* and *L. serriola*, respectively, explaining 25% and 16% of the PV. Both of these QTL co-located with those for AO potential from FRAP. An additional QTL for chlorophyll a was detected on LG9, explaining 12% of the variation for this trait and with allele inheritance from *L. sativa*, but in contrast to the previous QTL, this did not co-locate with AO potential.

#### Phenolic compounds

In both the field and glasshouse studies a single QTL for total phenolic content was identified. Each explained over 30% of the PV but were found on different LGs (8 and 4, respectively). QTLs were identified for 18 out of the 23 metabolites detected in the field grown RILs (excluding CTA1, CQA1, unknown metabolite 347, 5-CoQA1 and 5-CoQA2) and explained between 10% and 33% of the PV. QTLs were detected for 19 of the 23 phenolic compounds measured in the glasshouse grown RILs (with the exception of Q-3MG-7Gc, the two uncharacterised metabolites and 5-CoQA1). Individual QTL explained between 8% and 47% of the PV.

#### QTL hotspots

Almost every LG harboured regions of co-locating QTL (where LOD intervals overlapped), here defined as ‘QTL hotspots’, some of which were for the same trait measured in the two different growing environments (Supplementary Table S5). QTL for four phenolic compounds, DCTA, CTA1 and CTA2 in the glasshouse grown RILs and K-3MG in the field grown RILs were found to co-locate on LG1. QTL for the quercetin metabolite Q-3Gc on LG2 was found to co-locate with 3,5-DCQA and mDCTA from the glasshouse grown RILs. A similar cluster of QTL was identified on LG5, with a Q-3G measured from the glasshouse grown RILs co-locating with CMA and CTA1 measured from the field grown RILs. QTLs for CTA1 measured from the field and glasshouse trials were found to co-locate on LG5 and LG6 and a QTL for total phenolics measured from the field trial co-located with 5-CoQA.

LG3 contained the highest number of QTL (19 detected) and also a QTL hotspot of several individual compounds, which mapped to the centre of the LG within a 13 cM range. This included two kaempferol derivatives, from the glasshouse trial (K-3Gc) and the field trial (K-3MG), and a quercetin derivative from the glasshouse trial (Q-3MG). This region also corresponded to the large effect AO potential QTL plus QTL for total chlorophyll and carotenoids (Supplementary Table S5). This suggests that this region of the genome may be worthy of further investigation and development of molecular markers for breeding.

Each of the four QTL detected for AO potential measured in the glasshouse grown RILs were found to co-locate with other traits. In two instances, QTL for AO potential (on LG3 and LG7) were found to co-locate with QTLs for total chlorophyll and carotenoids (Supplementary Table S5). On LG4, the QTL for AO potential co-located with another for kaempferol derivative K-3MG also measured from the glasshouse grown RILs. On LG3 and LG9, QTL for AO potential co-located with QTL for Q-3MG, from the field and glasshouse trials, respectively. A QTL for Q-3MG-7Gc measured from the glasshouse RILs was also found to co-localise with the QTL for AO potential on LG9 (Supplementary Table S5).

### Identification of candidate genes

For the large effect AO QTL on LG3, candidate genes in the genomic region were identified in the lettuce reference genome sequence and their putative functions inferred, based on sequence similarity to the annotated genomes of *Arabidopsis thaliana* and *Solanum lycopersicum*. A total of 285 genes were identified from approximately 50 Mbp of the genome corresponding to the QTL region. Several genes acting in the phenylpropanoid pathway and known to directly influence secondary metabolism were identified within this QTL (Table 2). A gene which acts in the flavonoid biosynthetic pathway, flavanone 3-hydroxylase (F3H), was found within this region, which encodes a key enzyme in the synthesis of flavonoids quercetin and kaempferol.^48^

**Figure 5:**
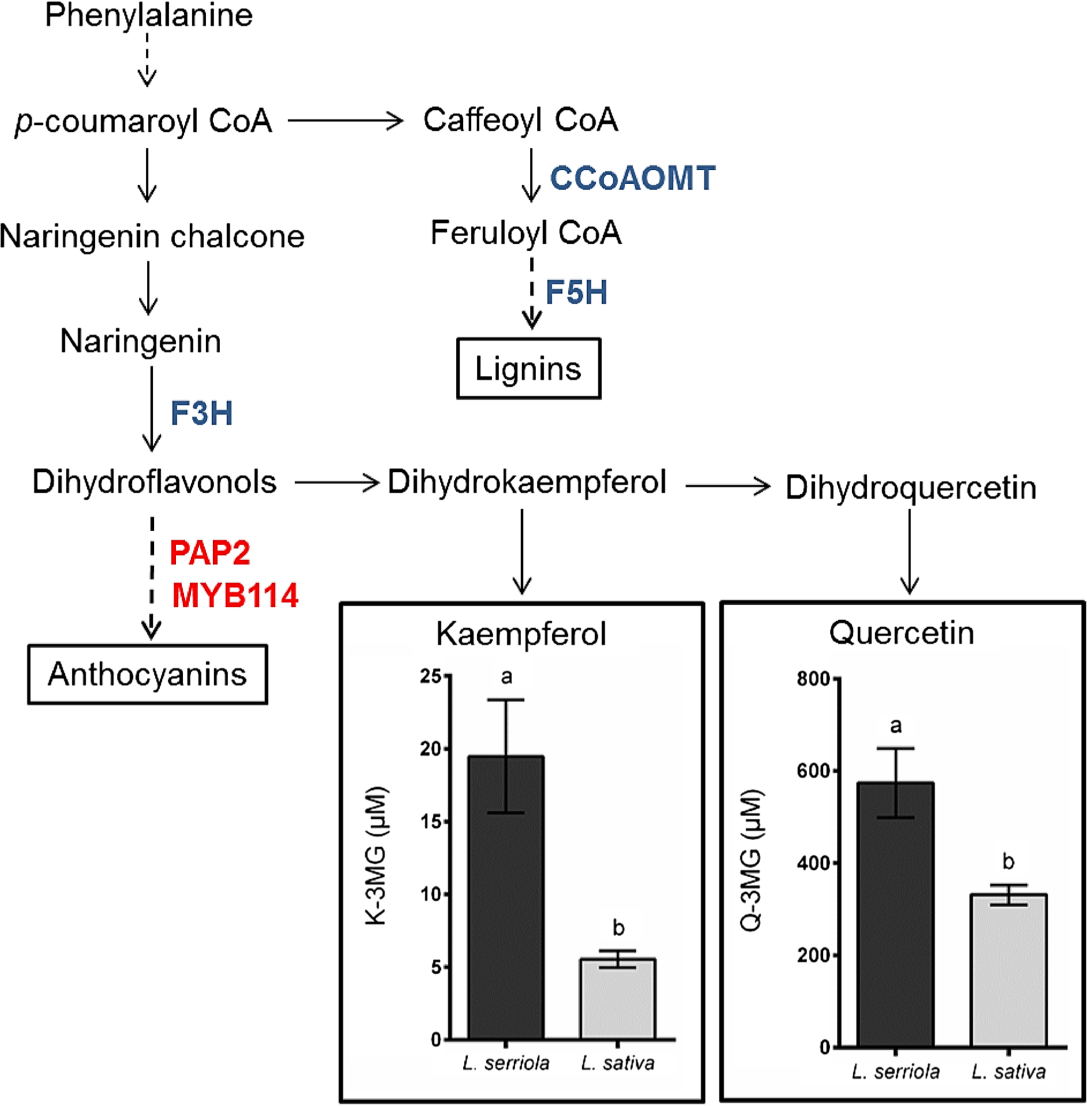
Candidates genes involved in the phenylpropanoid pathway. A summary of the phenylpropanoid pathway detailing roles of the candidate genes identified within the LG3 QTL region. Genes encoding three enzymes were identified: F3H, flavanone 3-hydroxylase; CCoAOMT, caffeoyl-CoA O-methyltransferase; F5H, ferulate-5-hydroxylase, coloured in blue and two MYB family transcription factors: PAP2; PRODUCTION OF ANTHOCYANIN PIGMENT 2 and MYB114, coloured in red. Differences in concentration of K-3MG and Q-3MG in wild and cultivated lettuce are shown. Letters indicate where significant differences were observed, as measured by two-sample *t*-test (*t*_5_ = 4.03, *P* ≤ 0.01, and *t*_5_ = 3.58, *P* ≤ 0.05, respectively).

**Figure 6:**
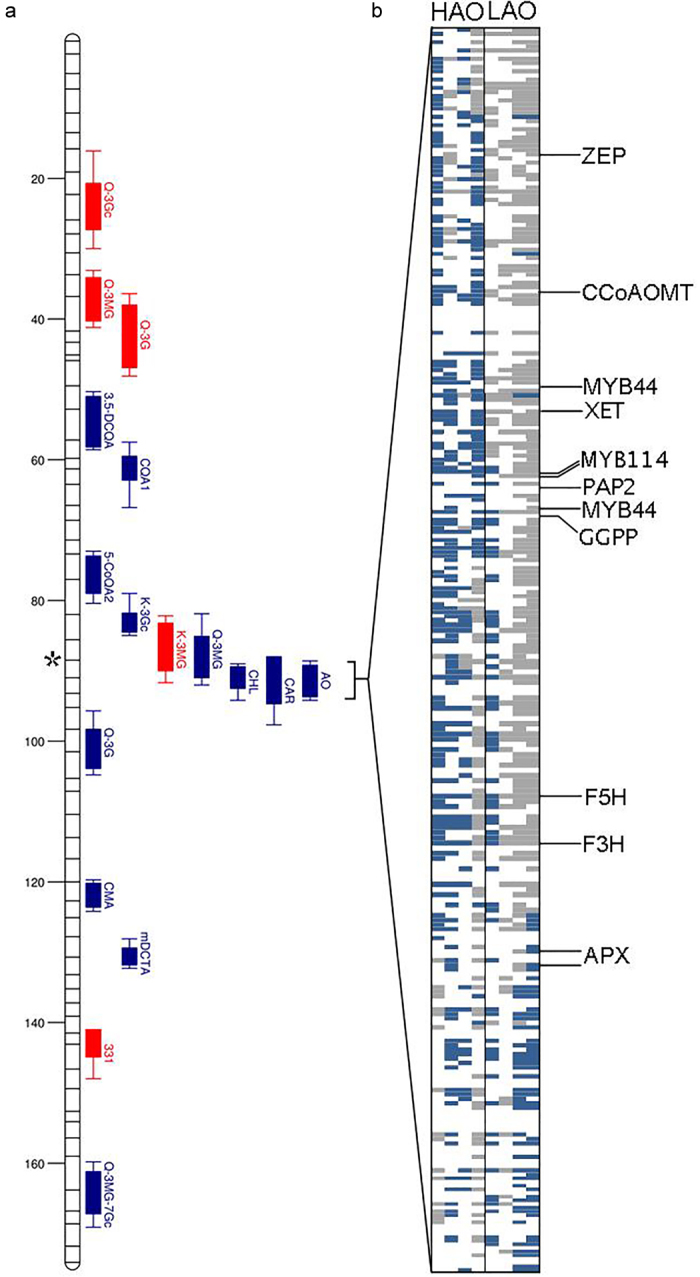
SNP genotyping of gene candidate under the LG 3 QTL or the AO QTL hotspot on LG 3 (**a**) SNP allele inheritance of the selected high antioxidant (HAO) RILs (columns 1–4) and low antioxidant (LAO) RILs (columns 5–8) was determined (**b**). Wild parent SNP allele inheritance is denoted in blue, inheritance of the cultivated parent allele is in grey and white boxes show where there is either missing sequencing data or no target SNPs present. Each row indicates one of the 285 candidate genes identified within the QTL region, highlighting the positions of the candidates described in Table 2. Position of the QTL hotspot is denoted by *with the scale bar to the left of the LG image showing genetic distance in centimorgans. Filled bars indicate the one-LOD interval and the line extensions show the two-LOD interval, for QTL identified in the field trial (blue) and glasshouse trial (red).

Two other enzymes acting in the phenylpropanoid pathway were identified (Figure 5); caffeoyl-CoA O-methyltransferase (CCoAOMT) which is involved in lignin biosynthesis^49^ and ferulate-5-hydroxylase (F5H), which is also involved in lignin biosynthesis but has recently been implicated in inducing the biosynthesis of anthocyanins under photooxidative stress in Arabidopsis.^50^ Two MYB transcription factors, one of which is production of anthocyanin pigment 2 (PAP2) and a closely related R2R3 class MYB transcription factor MYB114, both known to regulate the conversion of flavonol precursors (dihydrokaempferol and dihydroquercetin) to anthocyanin precursors (anthocyanidins) in the flavonoid biosynthetic pathway, were also located in this region.^51,52^ (Table 2, Figure 6) PAP2 was positioned within the estimated AO QTL peak and three distinct copies of MYB114, each spaced >20 kbp apart were in this region.

Genes encoding zeaxanthin epoxidase and geranylgeranyl pyrophosphate (GGPP) synthase, two genes involved in carotenoid biosynthesis,^53,54^ were also detected in this region (Table 2). Other notable candidates include a gene encoding ascorbate peroxidase (APX), involved in reactive oxygen species metabolism,^55^ a xyloglucan endotransglucosylase/hydrolase (XTH) involved in cell wall modification,^56^ and another MYB transcription factor (*MYB44*), found to delay leaf senescence when overexpressed in *A. thaliana.*^57^ Candidate gene analysis therefore revealed several genes which warrant further functional investigation.

For each of the 285 candidate genes identified within the AO QTL on LG3, inheritance of SNPs in coding regions for a selection of 8 RILs measured to have amongst the highest and lowest AO potential was determined by aligning genomic reads of *L. serriola* (UC96US23) with those of the RILs (Figure 6). SNP haplotype of the RILs was generally as expected, with the HAO RILs inheriting the wild parent SNP allele and LAO RILs having the cultivated parent allele and this was particularly pronounced in the region corresponding to the peak of QTL, which contained *PAP2* and *MYB114*.

### Analysis of candidate genes

**Figure 7:**
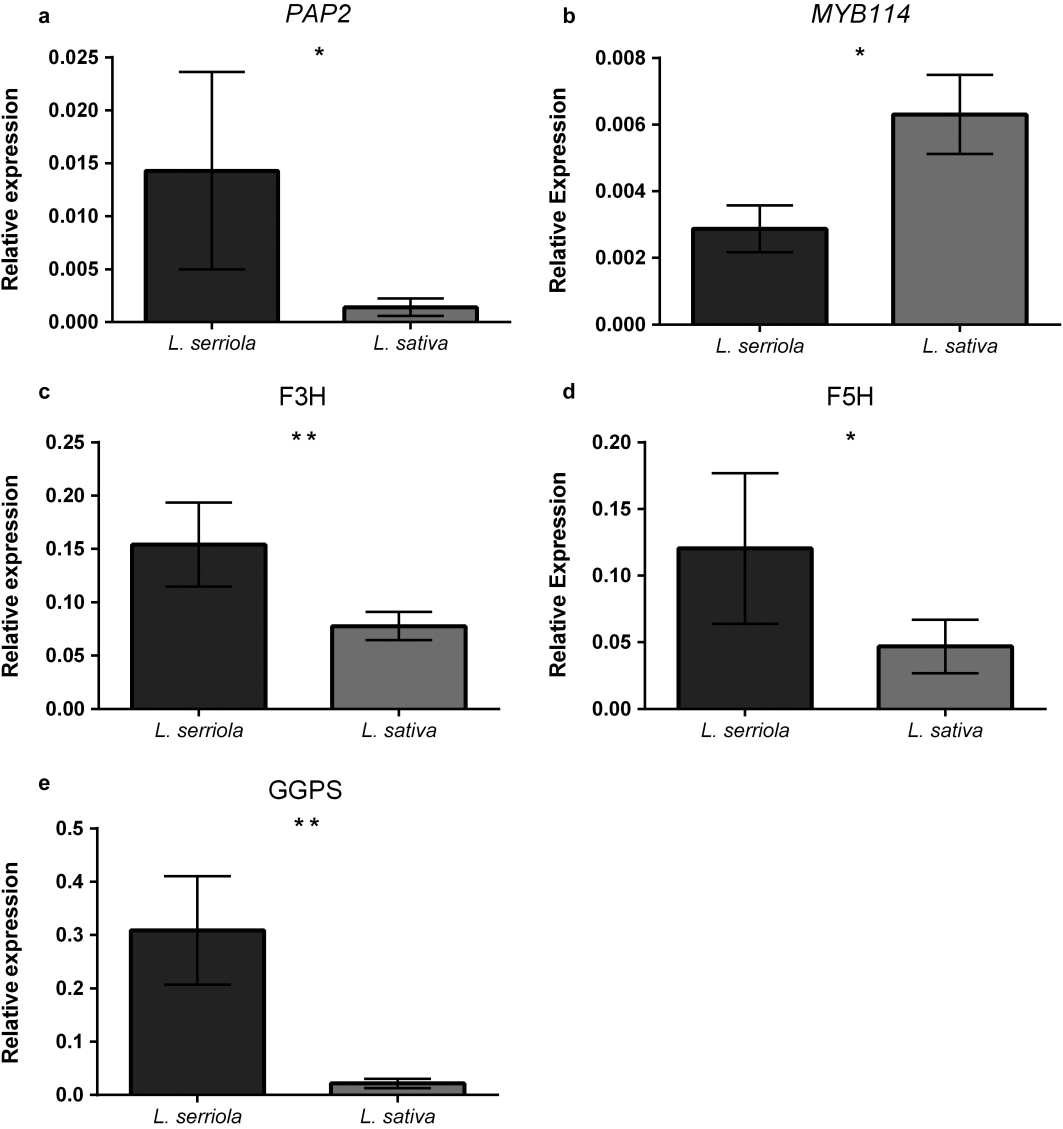
Comparison of candidate gene expression in wild and cultivated lettuce. Quantitative RT-PCR expression analysis of gene candidates detected within the AO QTL on LG3. PAP2 (**a**), MYB114 (**b**), F3H (**c**), F5H (**d**) and GGPS (**e**) were determined to be differentially expressed between wild (*L. serriola*) and cultivated (*L. sativa*) lettuce, with level of significance denoted as *P <0.05, **P <0.01. Bars represent mean ± standard error. Five remaining candidates which were not differentially expressed can be viewed in Supplementary Figure S2.

For the 10 candidates selected (Table 2), relative gene expression for both cultivated and wild parents was determined by qRT-PCR, in an attempt to identify differential expression. Five out of the 10 candidate genes were found to be differentially expressed between the parents (Figure 7), including *PAP2* (A, *P* < 0.05), *MYB114* (B, *P* < 0.05), *F3H* (C, *P* < 0.01), *F5H* (D, *P* < 0.05) and *GGPS* (E, *P* < 0.01) and are proposed as the best candidate genes from the 10 originally selected. With the exception of *MYB114,* all were more highly expressed in the wild parent. Three of these genes, *PAP2*, *MYB114* and *GGPS*, were located within the estimated QTL peak.

Comparison of the *L. sativa* cv. Salinas and *L. serriola* cDNA sequences revealed a number of non-synonymous amino acid changes in *MYB114*, including a seven amino acid deletion in the cultivated parent protein sequence (A, Supplementary Figure S3). The cultivated F3H protein had one amino acid difference from valine to isoleucine in comparison to the wild protein (B, Supplementary Figure S3) and F5H had three non-synonymous differences (C, Supplementary Figure S3). The *L. sativa* APX protein had four amino acid differences from *L. serriola* (D, Supplementary Figure S3).

## Discussion

### Understanding AO potential in lettuce

Our investigations of AO potential of a lettuce RIL population showed that phenolics, carotenoids and chlorophyll were important contributors to this quantitative trait, with evidence of transgressive segregation, perhaps indicating the complementary action of alleles inherited from both parents.^58^ Such lines offer exciting prospects for the development of lettuce with enhanced nutritional value. Transgressive segregants for fresh weight of tomatoes,^59^ milling quality in rice,^60^ aluminium tolerance in sorghum^61^ and grain yield in durum wheat^62^ have been proposed as potential sources for the improvement of these quantitative traits.

The phenolic composition of both wild and cultivated lettuce contrasted both qualitatively and quantitatively, with relative abundances differing amongst the RIL parents and with wild lettuce containing higher overall concentration of phenolic compounds. The greatest differences were observed for derivatives of CTA, caffeoyl quinic acid (CQA), quercetin-3-glucuronide (Q-3GC) and quercetin-3-malonylglucoside (Q-3MG; peaks 1, 3, 9 and 10; Figure 2), which were present in trace amounts or at greatly reduced levels in the cultivated parent. These dramatic differences are likely to significantly compromise the nutritional quality of the cultivated lettuce in comparison to its wild counterpart, subsequently impacting on associated health benefits following consumption. Metabolites such as CQA are lost or broken down when cooked,^63^ making lettuce an important source of these phenolics in the diet given that it is consumed raw and so even slight changes in metabolite abundance will have a major impact on health. DCTA(chicoric acid) was the most abundant phenolic in the RILs, consistent with other investigations measuring the phenolic composition of lettuce cultivars,^18,19^ with lettuce recognised as being one of the main European dietary sources of chicoric acid.^63^ Kaempferol derivatives were the least abundant phenolics detected, which are usually measured in trace amounts in comparison to other flavonols such as quercetin,^64^ a derivative of which (Q-3MG) was the second most abundant phenolic in both the field and glasshouse grown RILs.

Differences in phenolic concentrations were also observed amongst the RILs according to AO status. Increased concentrations of the most abundant phenolics such as chichoric acid, quercetin derivatives Q-3MG and Q-3G, CQA, CTA, DCQA, L-7G and K-3MG were observed in the HAO lines, though out of these metabolites only CQA and K-3MG were found to be significantly positively correlated with AO potential. The lack of significant correlation between AO potential and total phenolics (Table 1), despite the positive relationship observed for the extreme HAO and LAO RILs (Figure 4), is likely to reflect the wide genetic background of the RIL population.

### QTL for AO potential co-locate with numerous metabolites

For the first time to our knowledge, we have linked genomic regions in lettuce underlying AO status to candidate genes, using genomic resources developed for lettuce.^30^ In the present investigation, two QTL for total carotenoid content were identified on LG3 and LG7, which based on current literature are the only QTL so far determined for carotenoid content in a leafy vegetable. Although QTL for chlorophyll have previously been determined to vary depending on growing environment,^37^ QTL for chlorophyll content on LG3, 7 and 9 measured from the glasshouse trial confirm those previously identified from a UK field trial,^32^ providing strong evidence for consistency in these QTL. The four QTL identified for AO potential mapped to LG3, 4, 7 and 9, with the largest effect QTL on LG3 (LOD score 8.7) accounting for almost one-third (30.2%) of the PV for this trait, thus a large-effect QTL. Alleles inherited from *L. serriola* increased AO potential for all QTLs excluding alleles at the QTL on LG7, which was inherited from *L. sativa* in the majority of RILs with a higher AO potential. This was to be expected given that the wild parent was measured to possess an overall greater AO potential than cultivated lettuce.

It is perhaps unsurprising that QTL for total chlorophyll and carotenoids were found to co-locate on LG3 and 7, given their coordinated synthesis and intimate relationship in the chloroplasts as part of photosynthetic complexes^65,66^ and as the biosynthetic pathways are commonly linked through the precursor GGPP.^67^ GGPP was one of the candidates identified in the LG3 hotspot region (Table 2) which was found to be more highly expressed in the wild parent and could explain the co-location of QTL for chlorophyll and carotenoids observed. The co-locating QTL on LG7 for total chlorophyll and carotenoids explained 16–18% of the PV and possession of the *L. serriola* allele was found to increase trait values, which was expected as the wild parent had significantly increased concentrations of both pigments in comparison with *L. sativa*. QTL for total chlorophyll and carotenoids on LG3 had effects in the opposite direction as would be predicted with respect to the phenotype and measured gene expression, with the *L. sativa* allele increasing trait value. *Trans* arrangement of positive alleles has previously been linked with transgressive segregation of traits from an inter-specific cross of tomato; ^59^a phenomenon which was observed for both chlorophyll and carotenoids in the present investigation of lettuce. Co-location of QTL for total chlorophyll and carotenoids on LG3 and LG7 with total AO potential supports the findings of Hayashi *et al.*^37^ Despite this, although chlorophyll and carotenoid were positively correlated with each other, they were measured to be negatively correlated with AO potential in the present investigation. The large effect QTL for AO on LG3 (30.2% variation explained) also co-located with a QTL for Q-3MG, which explained 17.8% of the PV. QTL for AO on LG9 (16.2 % variation explained) also co-located with Q-3MG (16.8% variation explained), as well as the quercetin metabolite Q-3MG-7G (14.8% variation explained). As quercetin metabolites act as powerful AOs,^68^ it is therefore likely that fluctuations can notably affect total AO potential, suggesting that we have identified an important metabolic trait underpinning AO potential in this lettuce mapping population.

On each LG there was evidence of co-location of QTL for metabolites and in some cases, QTL for the same trait mapped to the same position in both environments. For example, QTL consistent across environments included CTA (CTA1) and on another LG, QTL for CTA1 and chicoric acid (DCTA) measured from the field trial co-located with a QTL for the kaempferol derivative K-3MG (kaempferol-3-malonylglucoside) measured from the glasshouse (Supplementary Table S5). Total phenolics measured from the field grown RILs co-located with 5-CoQA2 (5-*p*-coumaryl quinic acid isomer 2) measured from the glasshouse, which has a key role in the phenylpropanoid pathway for secondary metabolism biosynthesis.^69^ It has been known for some time that genes with a related function often cluster into operons in bacteria and there is growing evidence for the clustering of genes encoding secondary metabolites in plants.^70^ For example, metabolic gene clusters for terpenoid biosynthesis have now been found in oat and *Arabidopsis* and more recently in the wild legume *Lotus japonicas,*^71^ which may explain the many instances of QTL for different metabolites co-locating to the same region. QTL which have a consistent effect across different growing environments are considered more stable, thus are valued for use in breeding,^72^ but this was not observed for all traits. Given that only a subset of the total RIL population was used for this study, the ability to detect small effect QTL was likely to be limited as population size has been demonstrated to limit the sensitivity of QTL detection.^73^ Another possible explanation is that significant genotype × environment (G × E) interactions are occurring, which is perhaps unsurprising given that environmental factors are known to have an impact on secondary metabolism.^74^ Indeed, significant G × E interactions have been detected for AO and CHL QTL^37^; however, similar analyses of data collected from the glasshouse and field environments in present investigations would not be appropriate due to differences in experimental design.

Interestingly, a cluster of QTL co-located to the centre of LG3 for dry weight following nutrient limitation and drought recovery using this population,^75^ indicating a potential link between abiotic stress and AO potential, though direct comparisons of QTL were not possible due to differences in linkage maps utilised. Future work to analyse phenotype data using compatible mapping resources could reveal co-locations of QTL for abiotic stress tolerance and nutritional quality to the same genomic position, highlighting a strong target for marker-assisted breeding.

### Identifying candidate genes for AO potential

Using the Lettuce Genome Resource, along with the previously sequenced genomes of *A. thaliana* and *S. lycopersicum*, several promising gene candidates explaining variation in AO potential in the lettuce RILs were identified on LG3, including two MYB transcription factors (*PAP2* and *MYB114*) thought to regulate anthocyanin biosynthesis (Table 2). Expression analysis revealed that both *PAP2* and *MYB114* genes were differentially expressed (Figure 7), with expression increasing and decreasing, in the wild and cultivated parents, respectively. Anthocyanins are a subclass of flavonoids synthesised from dihydroflavonols known to be one of the major compounds controlling plant colour, particularly fruits and this is largely regulated by the MYB transcription factors.^51^ The presence of anthocyanins in the red lettuce cultivars investigated (C1 and C2; Figure 4) is likely to have resulted in a higher AO and phenolic content compared to the green cultivars and the high AO RILs, the latter of which are green-leaved, with no anthocyanin metabolites detected in this population. Mulabagal *et al.*^23^ investigated the phenolic contents of red and green lettuce types and although one anthocyanin was identified in red lettuce (cyanidin-3-*O*-(6’’-malonyl-b-glucopyranoside)), none were detected in green types, which is also supported by phenolic composition analysis by Llorach *et al.*^19^ Enhancing the expression of *PAP1* and the highly similar *PAP2* (93% identity of the R2R3 domain) using activation-tagging in *Arabidopsis* resulted in increased expression of phenylpropanoid biosynthesis genes, including phenylalanine ammonia lyase, the enzyme which initiates the phenylpropanoid pathway and chalcone synthase, the first enzyme acting in flavonoid biosynthesis,^76^ which could explain how the increased expression of PAP2 observed could contribute to AO status, despite the lack of anthocyanins detected. MYB114 also has a role in regulating anthocyanin biosynthesis that is similar to PAP2, through interaction with basic helix-loop-helix (bHLH) proteins, in a mechanism which is highly conserved throughout the plant kingdom.^77^ Given that effects of *MYB114* overexpression are dependent on overexpression of a corresponding bHLH transcription factor, this could explain how *L. sativa* had reduced phenolic content and AO status compared to *L. serriola*, despite exhibiting increased expression of *MYB114*. Synchronised increases in the expression of MYB and bHLH transcription factors may result in the red leaf phenotype observed in commercial lettuce types.

Another promising gene candidate identified within this region was *F3H*, which was more highly expressed in wild relative to cultivated lettuce. F3H is involved in the conversion of naringenin to the dihydroflavonols dihydrokaempferol and dihydroquercetin (Figure 5), which are the precursors for kaempferol and quercetin, respectively.^48^ Both kaempferol and quercetin were present in higher concentrations in the wild parent than the cultivated parent (Figure 5), likely to result from an increased abundance of dihydroflavnols caused by increased levels of F3H and consistent with the former having a greater AO potential. Dihydroquercetin is essential not only as a precursor for quercetin metabolites, but also for flavonoids such as catechin and the proanthocyadins,^78^ which may also contribute to AO potential. Derivatives of quercetin (Q-3MG-7Gc) and kaempferol (K-3MG) were found to be strongly positively correlated in the present investigation (Table 1), indicating tightly coordinated regulation of the biosynthesis of these flavonoids.

Ferulate-5-hydroxylase (F5H), an enzyme acting in the phenylpropanoid biosynthesis pathway,^79^ was also found to be more abundant in wild lettuce. Knocking out *F5H* in *Arabidopsis* has revealed a range of phenotypes, affecting lignin biosynthesis, UV protection and response to wounding.^80^ *F5H* mutants had increased expression of *MYB4,*^80^ a negative regulator of chalcone synthase,^81^ thus reducing flavonoid biosynthesis which is consistent with the reduced levels of flavonoids detected in cultivated lettuce in the present investigation.

### Sustainable intensification and breeding for increased AO potential

Enhanced food security requires that we achieve ‘more from less’ and that yield enhancements in future crops must be complemented by higher nutritional value (Agri-tech Strategy, www.gov.co.uk). Many crop-breeding programmes are now dedicated to developing enhanced crop nutrition where wild progenitors of crops may be exploited for higher concentrations of target phytonutrients relative to those observed in their commercial counterparts.^82,83^ Indeed, such an approach has been successfully deployed for many food crops including tomatoes,^34^ berries^84,85^ carrots^86^ and potatoes.^87^ This can be a powerful approach – broccoli florets from cultivated varieties were found to have between 3 and 10 μmol/g of health-benefitting glucosinolates, whilst wild species can contain 50–100 μmol/g.^83^

Here, the AO potential of cultivated lettuce (*L. sativa* cv. Salinas) was significantly lower than that of the wild progenitor (*L. serriola*), with notable differences in phenolic composition. Past artificial selection of lettuce for improved yield traits is likely to be linked to indirect selection against characteristics such as AO status since phenolics are known to have a bitter taste.^88^ During evolution we have learned to reject bitter tastes and with >50 bitter taste receptors characterised, aversion is likely to have been crucial to survival.^88^ A RIL (HAO3) which had comparable levels of AOs to the red varieties but a reduced phenolic content was identified in the present investigation (Figure 4), which could be utilised in future breeding programmes.

Co-incidentally, improving leaf nutritional quality may also afford greater plant protection from pests and diseases, given that many secondary metabolites have roles in defence against herbivore and pathogen attack^89^ with mechanical wounding resulting in the accumulation of phenolic compounds in lettuce.^90^ Microbial spoilage in particular has been shown to reduce the shelf life of lettuce.^91^ Tomatoes genetically engineered to overexpress anthocyanins had an increased shelf life in comparison to wild-type-cultivated tomatoes, with the transgenic tomatoes demonstrating reduced susceptibility to the fungal pathogen *Botrytis cinerea*.^92^ Leaf AO potential and derivatives of the phenolics, quercetin and kaempferol, measured in present investigations were found to positively correlate with shelf life, measured on the same RIL subset by Zhang and colleagues,^32^ further supporting a link between phytochemicals and shelf life. A gene encoding a XTH involved in cell wall loosening and located within the QTL hotspot in LG3 has also been linked with shelf life, with the down-regulation of a XTH previously shown to increase the shelf life of lettuce,^93^ though the XTH identified was not determined to be differentially expressed between the wild and cultivated parents in present investigations (Supplementary Figure S2). Clearly further investigations are required to dissect the link between nutritional quality and shelf life in lettuce.

Improving the phytonutrient content of widely consumed yet relatively nutritionally poor vegetables, such as lettuce, is an important target for plant breeding and here we identified several candidates controlling flavonoid biosynthesis within the large-effect QTL for AO potential; a number of which were shown to be differentially expressed between wild and cultivated lettuce. The QTL region underpinning these traits is a strong target for future breeding and on-going research is focusing on introgressing this genomic region into commercial lettuce breeding lines. At the same time, further proof of functionality through genome editing and other molecular routes is also underway. Taken together, this study provides the first detailed insight into lettuce phytonutrient traits and how they may be deployed in the future for an enhanced food plant, consumed widely and of global significance.

## Figures and Tables

**Table 1:** Pearson’s correlation coefficient analysis of traits measured from the RIL population.

Trait	Shelf life	AO	Phenolics	CHL	CAR	Q-3MG-7Gc	K-3MG	CQA2	CQA3	5-CoQA2
Shelf life	1									
AO	0.304*	1								
Phenolics	−0.115	0.199	1							
CHL	−0.254	−0.414**	−0.298	1						
CAR	−0.275*	−0.402**	−0.277	0.987	1					
Q-3MG-7Gc	0.417*	0.434*	0.309	−0.181	−0.181	1				
K-3MG	0.433*	0.409*	0.042	0	0.002	0.648***	1			
CQA2	0.184	0.461**	0.157	−0.062	−0.062	0.505**	0.209	1		
CQA3	0.264	0.439*	0.354	−0.14	−0.14	0.634***	0.396*	0.911***	1	
5-CoQA2	0.005	0.384*	0.471**	−0.162	−0.165	0.504**	0.475**	0.416*	0.49**	1

Correlation of mean trait values measured in the glasshouse trial, with shelf life data taken from previous investigations on the same RIL subset, taken from Zhang *et al.* (ref. 32). ***P < 0.001; **P < 0.01 and *P < 0.05 levels. AO, antioxidant potential; CHL, total chlorophyll content; CAR, total carotenoid content. Correlations for all traits measured can be seen in Supplementary Table S3.

**Table 2:** Candidate genes identified within the AO QTL on LG3.

*A. thaliana* ccession	*Lactuca* accession^a^	Functional description	Reference
At1g66390	Letassy_X1_8017	Production of anthocyanin pigment 2 (PAP2) protein	51
	Serrassy_T_P2_17469	R2R3 class of MYB transcription factors, involved in anthocyanin biosynthesis	
AT1G66380	Letassy_X1_6767	MYB114 R2R3 class of MYB transcription factors closely related to the production of anthocyanin pigment type MYBs involved in anthocyanin biosynthesis	50
	Serrassy_T_P2_17850		
At3g51240	Letassy_X1_4796	Flavanone 3-hydroxylase, enzyme involved in flavonoid biosynthesis	47
	Serrassy_T_P2_12444		
At4g36220	Letassy_X1_2126	Ferulate-5-hydroxylase, enzyme involved in lignin and anthocyanin biosynthesis	49
	Serrassy_T_P2_6166		
AT4G34050	Letassy_X1_23118	Caffeoyl-CoA O-methyltransferase, enzyme involved in lignin biosynthesis	48
	Serrassy_T_P2_7154		
AT4G36810	Letassy_X1_21865	GGPP synthase, enzyme involved in terpenoid (includes carotenoids) biosynthesis	52
	Serrassy_T_P2_6921		
At5g67030	Letassy_X1_1094	Zeaxanthin epoxidase precursor, enzyme involved in zeaxanthin (common carotenoid) biosynthesis	53
	Serrassy_T_P2_6429		
AT5G65730	Letassy_X1_23118	XTH 7, regulated by MYB41 and has a putative role in cell expansion	55
	Serrassy_T_P2_7154		
AT5G67300	Letassy_X1_21649	MYB44 R2R3 class of MYB transcription factor found to have multiple roles in ABA signalling and leaf senescence	56
	Serrassy_T_P2_5693		
AT4G35000	Letassy_X1_9546	Ascorbate peroxidase, enzyme involved in reactive oxygen species metabolism in leaf peroxisomes	54
	Serrassy_T_P2_46272		

a Both *L. sativa* and *L. serriola* accessions tabulated.

## Notes

GJJC and SDR are employed by the company Vitacress Ltd, providing financial support for this research.
