# Supplementary material for: Elucidating the genetic basis of antioxidant status in lettuce (Lactuca sativa)
Source: Hortic Res. 2015 Nov 25;2:15055–. doi: 10.1038/hortres.2015.55 (PMC4660231; doi:10.1038/hortres.2015.55)
Supplement: Supplementary Information [file hortres201555-s1.docx]

**Supplementary METHODS***Determination of antioxidant potential*Research by Payne *et al.,* (ref. 39) has confirmed that the Ferric Reducing Antioxidant Power assay (FRAP assay; refs. 38-39) gives qualitatively similar data to that of ORAC (Oxygen Radical Absorbance Capacity), which is often used to measure antioxidant activity in food quality research. To prepare samples for the assay, ground leaf material was taken from -80°C storage and centrifuged (5 min, 13,000 rpm) in QiaShredder® tubes (QIAGEN, UK) to separate the aqueous fraction from the solid leaf material. The FRAP assay was carried out in flat-bottom 96-well microtitre plates and compared to blank samples of water and internal standards (0.25 – 6 mM) of iron sulphate heptahydrate (Fe^II^) were used to generate a standard curve. 10 μl samples of leaf extract, Fe^II^ standard or water were added to the wells of the plate. FRAP reagent was prepared fresh as required by combining 300 mM acetate buffer pH 3.6, 10 mM 2,4,6-tripyridyl-*s*-triazine in 40 mM HCl and 20 mM ferric chloride hexahydrate at a ratio of 10:1:1. 300 μl of FRAP reagent and 30 μl H_2_O were added to the samples and after 2 minutes optical density (OD) at 620 nm was measured using a plate-reading spectrophotometer (Anthos Labtec Instruments). The antioxidant capacity of samples were calculated using the standard calibration curve and was reported as Fe^II^ equivalents, mmol.g^-1^ fresh weight (FW). *Identification and quantification of individual phenolics*10 μl of each sample of total leaf phenol extract was injected onto a Phenomenex Synergi Hydro RP 80 A 150 x 2.0 mm 4 μm column. Phenols were separated in a two-eluent system, consisting of 0.1% (v/v) formic acid in ultrapure water (eluent A) and 0.1% (v/v) formic acid in methanol (eluent B). Eluents were pumped at a flow rate of 0.2 ml min^-1^ using the gradient: 0 min, 0% B; 30 min, 50% B: 32 min, 100% B; 34 min, 100% B; 40 min, 0% B; 45 min, 0% B. Phenols were detected and identified by a combination of (1) UV absorbance using an Acela PDA with traces collected at 280, 330 and 520 nm, and (2) MS in negative ion mode using an LCQ fleet mass spectrophotometer fitted with an electrospray ionisation interface. The MS detector was tuned against morin and data was collected using two scan events: (1) full scan analysis in the *m/z* range 80 – 2000, and (2) data dependent MS/MS using a collision energy of 35%. Compounds were identified using their UV absorption characteristics and parent and daughter ion masses as described (ref. 19) . Relative quantification was achieved from the parent ion peak area, corrected according to the peak area of the morin internal standard.

*Identification of candidate genes*To identify candidate genes underlying the traits analysed, contig sequences of *L. sativa* cv. Salinas and *L. serriola* UC96US23 containing the SFP markers flanking the 2-LOD confidence interval for QTL were identified (http://cgpdb.ucdavis.edu/cgpdb2/). Contig sequences were searched using the GBrowse tool accessible via the Lettuce Genome Resource (https://lgr.genomecenter.ucdavis.edu), to identify the larger genomic scaffolds in the QTL region. To identify gene models, genomic scaffolds were downloaded and the genes in the intervening region were identified in local BLASTn searches using BioEdit ^94^, against the publicly available and well annotated *Arabidopsis thaliana* genome and the more closely related *Solanum lycopersicum* genome (downloaded via <http://www.plantgdb.org/>). Gene sequences were searched via NCBI BLAST against the lettuce transcriptome shotgun assembly ^95^ to provide a *Lactuca* locus reference. A table including the top ten BLASTn hits for each of the ten candidate genes selected against the NCBI database can be viewed in Table S6.

*Quantitative RT-PCR*
Quantitative real time PCR was conducted to evaluate the expression of 10 candidate genes selected from the genomic regions underlying the LG3 AO QTL for the wild and cultivated parents of the mapping population. RNA was extracted from three biological replicates of both *L. sativa* cv. Salinas and *L. serriola* planted in the field experiment described, following a modified version of the CTAB protocol ^96^. CTAB extraction buffer containing 2% (w/v) CTAB (hexadecyltrimethylammonium bromide), 2% (w/v) PVP (polyvinylpyrrolidinone), 100 mM Tris-HCl (pH 8.0), 25 mM EDTA and 2 M NaCl was incubated at 65°C and 800 μl of buffer was added to approximately 300 mg of ground leaf tissue, along with 50 μl of 2-ME (2% β-mercaptoethanol). Samples were incubated at 65°C for 5 minutes, before adding 800 μl of 24:1 Chloroform : Isoamyl alcohol, mixing and centrifuging for 10 minutes at 13,200rpm, after which the aqueous phase was transferred to a fresh microcentrifuge tube. The Chloroform : Isoamyl alcohol step was then repeated and 180 μl of 10 M LiCl was added to the aqueous phase, before precipitating at 4°C for 18 hours. Extracts were centrifuged at 4°C for 15 minutes at 13,200rpm and the supernatant was discarded, before dissolving the pellet in 700 μl of pre-warmed SSTE buffer containing 1M NaCl, 0.5% (w/v) SDS, 10 mM Tris-HCl (pH 8.0) and 1mM EDTA for 5 minutes at 60°C. 700 μl of Chloroform : Isoamyl alcohol was then added, extracts were centrifuged at 13,200rpm for 10 minutes at 18°C and the aqueous phase was added to an equal volume of 100% ethanol and stored at -20°C for 30 minutes. The extract was then centrifuged at 4°C for 20 minutes at 13,200rpm and the RNA pellet was washed in 1ml of ice-cold 70% ethanol and allowed to dry, before dissolving in 50 μl DEPC-treated water (Sigma Aldrich, UK). Total RNA quality and concentration was estimated using a nanodrop (NanoDrop 1000 Spectrophotometer, Thermo Scientific), and to ensure A260/A280 ratio was ~2.

First strand cDNA was synthesised from 1 μg of total RNA by reverse transcription using the SuperScript III 1^st^ Strand qPCR SuperMix (Life Technologies, UK), according to the manufacturer’s guidelines. Concentration was estimated using a nanodrop and cDNA was diluted to 150ng/μl in DEPC-treated water for qPCR. Primer3 ^97^ was used to generate oligos for the expression of the ten gene candidates (Integrated DNA Technologies, UK), using the following parameters: GC content 40-60%, melting temperature 55-62°C, 16-25 bp (Table S3). Where possible, primers were designed to span exon:exon junctions, estimated by aligning *Lactuca* transcriptome sequences for each gene to the annotated *Arabidopsis* homologue. Five reference genes were tested for expression stability (Acitn; ACT, Elongation Initiation Factor gamma subunit; EIF2A, TAP42-interacting protein of 41 kDa; TIP41, UBC21, and eukaryotic small ribosomal subunit; 40S ^98^) using NormFinder ^99^ and ACT and 40S were selected for use in investigations.

The qPCR reaction was performed using the PowerUp SYBR Green Master Mix (Life Technologies), with 150 ng of cDNA and 3-5 pmol of each forwards and reverse primers in a 10 μl final reaction volume on a StepOnePlus Real-Time PCR System (Applied Biosystems, UK), under the following settings: 50°C for 2 minutes, 95°C for 2 minutes and 40 cycles of 5 seconds denaturation at 95°C, annealing for 10 seconds at 58°C and extension for 15 seconds at 72°C. Melting curve analysis was conducted immediately after the qPCR reaction from 60-95°C in 0.3°C increments to confirm the absence of primer dimers, DNA contaminants and secondary products. Three biological replicates of each parent were tested in duplicate for each gene target and two reference genes, along with duplicates of no template controls for each primer set.

PCR efficiency (E) was calculated for each individual reaction using LineRegPCR (available from: linregpcr.nl/) and an average efficiency for each primer set per plate was used in calculations ^100^. Relative expression was determined using the equation: R = (*E_B_* + 1)*^Cq(B)^* / *E_A_* + 1)*^Cq(A)^*, with *E* denoting primer efficiency for A; the target gene and B; reference gene and *Cq* denoting quantification cycle, described by ref.^101^, normalised to the geometric means of reference genes ACT and 40S ^102^.
