# Supplementary figures and images for: Elucidating the genetic basis of antioxidant status in lettuce (Lactuca sativa)
Source: Hortic Res. 2015 Nov 25;2:15055–. doi: 10.1038/hortres.2015.55 (PMC4660231; doi:10.1038/hortres.2015.55)

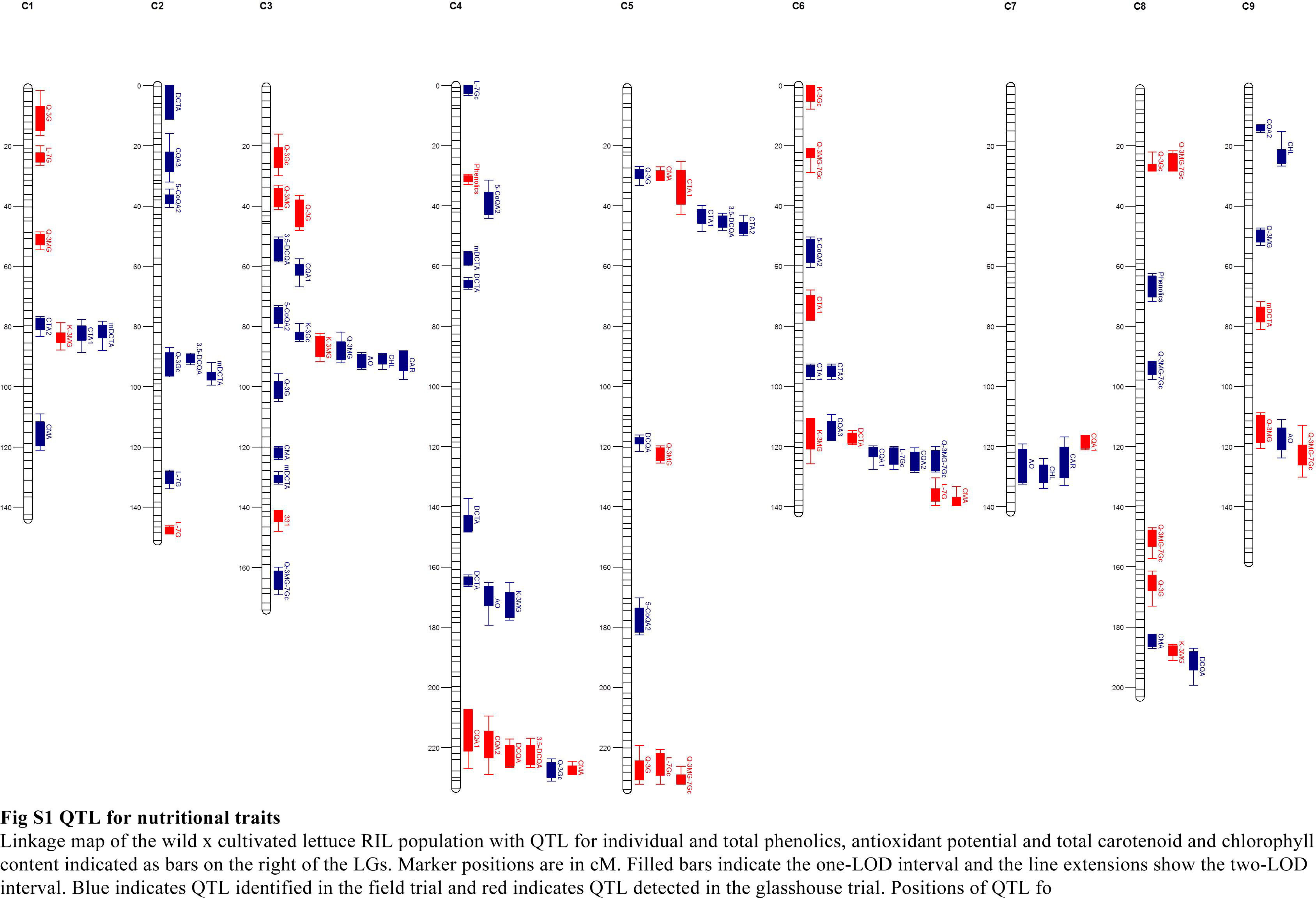

Supplement: Supplementary Figure S1 [file hortres201555-s2.jpg]

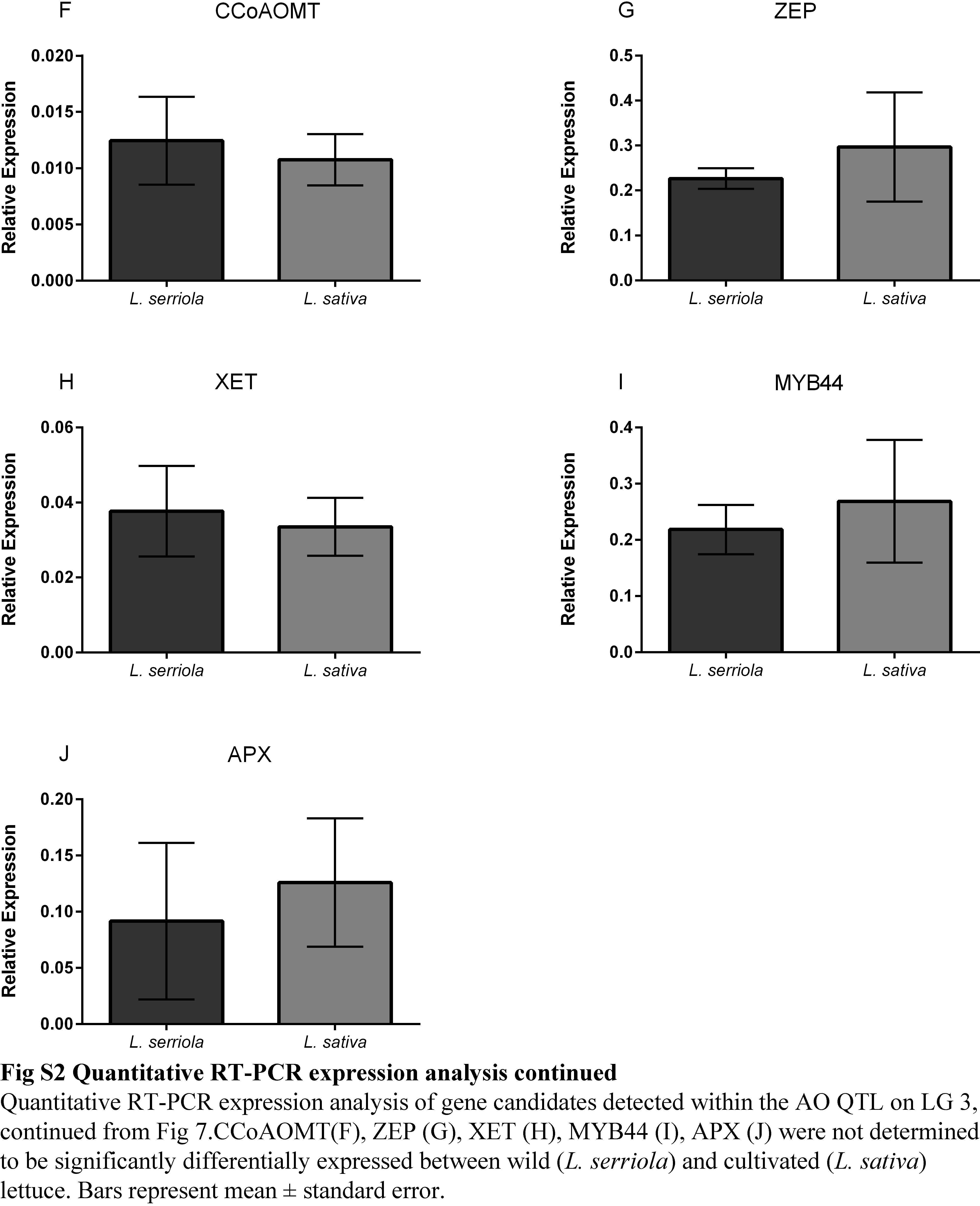

Supplement: Supplementary Figure S2 [file hortres201555-s3.jpg]

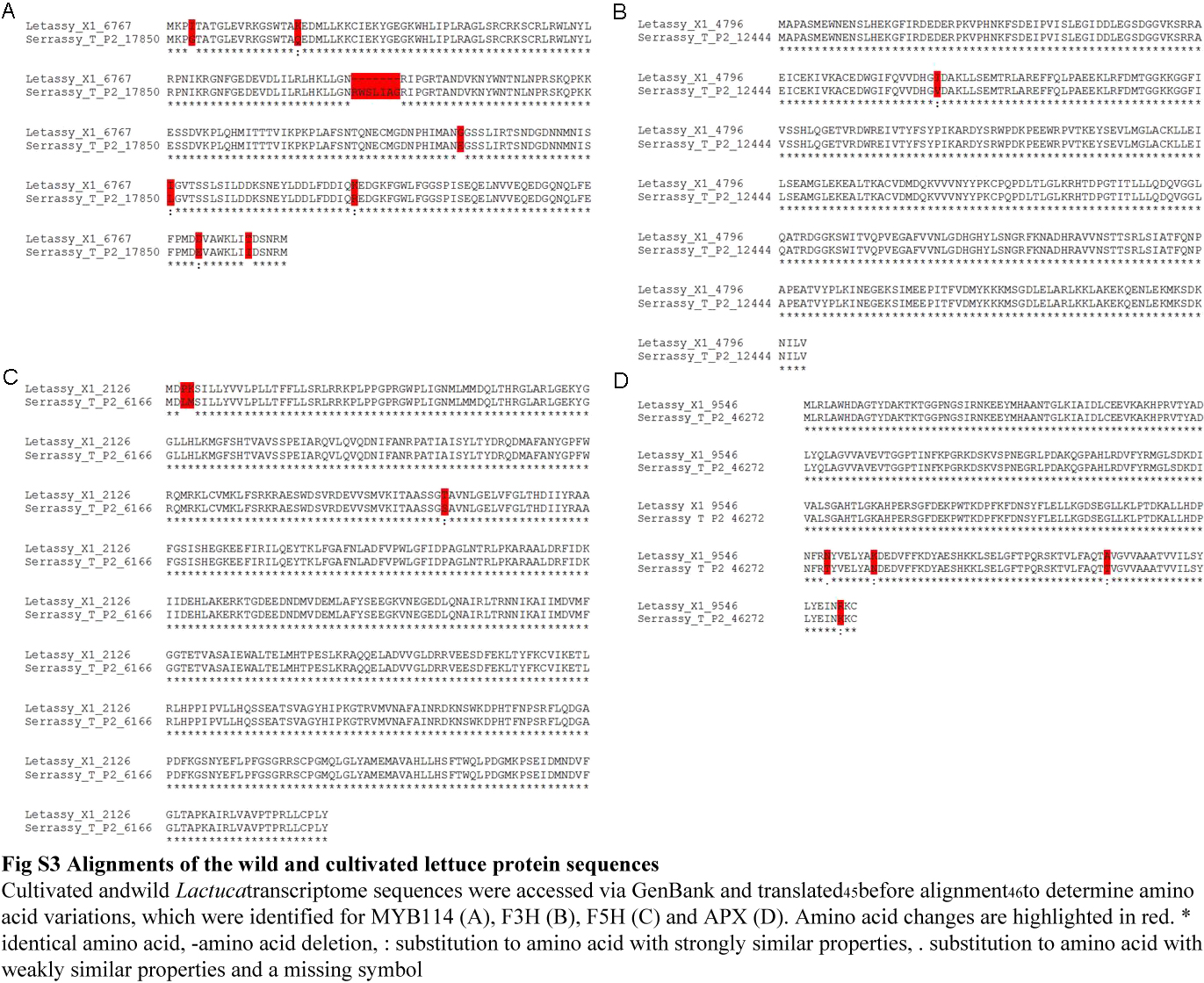

Supplement: Supplementary Figure S3 [file hortres201555-s4.jpg]
