# Supplementary material for: Elucidating the genetic basis of antioxidant status in lettuce (Lactuca sativa)
Source: Hortic Res. 2015 Nov 25;2:15055–. doi: 10.1038/hortres.2015.55 (PMC4660231; doi:10.1038/hortres.2015.55)
Supplement: Supplementary Table S3 [file hortres201555-s7.docx]

**Table S2 Primer sequences utilised for quantitative RT-PCR.**

| Gene symbol | Accession ^a^ | Primer sequence (5’-3’, forward/reverse) | RT-PCR efficiency ^b^ | Amplicon length (bp) |
| --- | --- | --- | --- | --- |
|  |  |  |  |  |
| ACT | AY260165 * | AGGGCAGTGTTTCCTAGTATTGTTG/ CTCTTTTGGATTGTGCCTCATCT | 1.98 | 106 |
| EIF2A | EU028334.1 * | TAGGCGAGTGGAGAAGCATT/ GTAGAAACAGCAACAGGCAAA | 1.92 | 71 |
| TIP41 | Lact_sati.cst1.6123 * | GAGAGATTTGCTGGAGGGAAACTA/ CCTTTGACTGATGATGTTTGGA | 1.90 | 101 |
| UBC21 | AT5G25760 * | TCTTAGATCACCGTCCCATCGT/  TCTGAGATTGTCCGAGGATATGAG | 1.93 | 89 |
| 40S | HS586765.1 * | CAAGATTCGGTGACAGGGATG/ CACCACCTCCAAATCCACCA | 2.00 | 137 |
| PAP2 | Serrassy_T_P2_17469 | GCATGGACTGCTGATGAAGA/ TGAAGCCTAAGCATGAGATCAA | 1.74 | 200 |
| MYB114 | Letassy_X1_6767 | GCAGGGTTAAGCAGATGCAG/ AAAAGCTTGTGAAGCCTGAG | 1.88 | 139 |
| F3H | Letassy_X1_4796 | GATGGTGGCAAGAGTTGGAT/ GAGCAGGGTTTTGGAATGTC | 2.07 | 191 |
| F5H | Letassy_X1_2126 | TGTCCTGGAATGCAACTTGG/ GCTTTTGGTGCAGTGAGTCCA | 2.01 | 142 |
| CCAoM | Letassy_X1_23820 | CTTGAAACCTCCGTCTACCC/  ATGGTGTTCTTGGCGTTGAT | 1.88 | 152 |
| GGPS | Letassy_X1_21865 | TTGATTTTTCGATCCCCAAC/ GGCTTTTGTTTCAGGTGGTG | 1.84 | 132 |
| ZEP | Letassy_X1_1094 | TGCATCACTGGTCAACGAAT/  TTTCTTCCCCAACAGCATCT | 1.73 | 166 |
| XET | Letassy_X1_23118 | TCCAGCCAATGGGAGTCTAC/  GCACCCCTCAATGTCAAAGT | 1.92 | 131 |
| MYB44 | Letassy_X1_21649 | GTCGGACTGATAACGCCATT/  ACGTCAGATCCAGACGGACT | 2.06 | 190 |
| APX | Letassy_X1_9546 | TATCTCCGCCGCTATTCATC/  GGCTCGGAGAAGCTAAGGAT | 1.76 | 150 |
|  |  |  |  |  |

^a^ GenBank database

^b^ Calculated by LinRegPCR program (ref. 35)

* Taken from ref. 49
