# Supplementary material for: Elucidating the genetic basis of antioxidant status in lettuce (Lactuca sativa)
Source: Hortic Res. 2015 Nov 25;2:15055–. doi: 10.1038/hortres.2015.55 (PMC4660231; doi:10.1038/hortres.2015.55)
Supplement: Supplementary Table S6 [file hortres201555-s10.docx]

**Table S6 Top ten BLASTn hits for the ten selected candidate genes against the GenBank database**

| **Gene  symbol** | **Rank** | **Description** | **Accession** |
| --- | --- | --- | --- |
| PAP2 | 1 | [*Gynura bicolor* GbMYB2b mRNA for transcription factor MYB2b, complete cds](http://blast.st-va.ncbi.nlm.nih.gov/Blast.cgi#alnHdr_685836931) | [AB581527.1](http://www.ncbi.nlm.nih.gov/nucleotide/685836931?report=genbank&log$=nucltop&blast_rank=1&RID=1Z7910KE01R) |
|  | 2 | [*Gynura bicolor* GbMYB2a mRNA for transcription factor MYB2a, complete cds](http://blast.st-va.ncbi.nlm.nih.gov/Blast.cgi#alnHdr_685836929) | [AB581526.1](http://www.ncbi.nlm.nih.gov/nucleotide/685836929?report=genbank&log$=nucltop&blast_rank=2&RID=1Z7910KE01R) |
|  | 3 | [*Gynura bicolor* GbMYB2 mRNA for transcription factor GbMYB2, complete cds](http://blast.st-va.ncbi.nlm.nih.gov/Blast.cgi#alnHdr_306922321) | [AB550245.1](http://www.ncbi.nlm.nih.gov/nucleotide/306922321?report=genbank&log$=nucltop&blast_rank=3&RID=1Z7910KE01R) |
|  | 4 | [*Gynura bicolor* GbMYB1 mRNA for transcription factor GbMYB1, complete cds](http://blast.st-va.ncbi.nlm.nih.gov/Blast.cgi#alnHdr_306922319) | [AB550244.1](http://www.ncbi.nlm.nih.gov/nucleotide/306922319?report=genbank&log$=nucltop&blast_rank=4&RID=1Z7910KE01R) |
|  | 5 | [*Dahlia pinnata* R3myb mRNA for transcription factor R3myb, complete cds](http://blast.st-va.ncbi.nlm.nih.gov/Blast.cgi#alnHdr_327165090) | [AB621921.1](http://www.ncbi.nlm.nih.gov/nucleotide/327165090?report=genbank&log$=nucltop&blast_rank=5&RID=1Z7910KE01R) |
|  | 6 | [*Gerbera* hybrid cv. 'Terra Regina' mRNA for MYB10 protein](http://blast.st-va.ncbi.nlm.nih.gov/Blast.cgi#alnHdr_40643885) | [AJ554700.1](http://www.ncbi.nlm.nih.gov/nucleotide/40643885?report=genbank&log$=nucltop&blast_rank=6&RID=1Z7910KE01R) |
|  | 7 | [*Pericallis cruenta* MYB10 mRNA, partial cds](http://blast.st-va.ncbi.nlm.nih.gov/Blast.cgi#alnHdr_379054200) | [JF499667.1](http://www.ncbi.nlm.nih.gov/nucleotide/379054200?report=genbank&log$=nucltop&blast_rank=7&RID=1Z7910KE01R) |
|  | 8 | [*Dahlia pinnata* DvMYB1 mRNA for R2R3-MYB transcriptional factor, complete cds](http://blast.st-va.ncbi.nlm.nih.gov/Blast.cgi#alnHdr_312222648) | [AB601003.1](http://www.ncbi.nlm.nih.gov/nucleotide/312222648?report=genbank&log$=nucltop&blast_rank=8&RID=1Z7910KE01R) |
|  | 9 | [*Chrysanthemum* x *morifolium* MYB10 mRNA, partial cds](http://blast.st-va.ncbi.nlm.nih.gov/Blast.cgi#alnHdr_379054202) | [JF499668.1](http://www.ncbi.nlm.nih.gov/nucleotide/379054202?report=genbank&log$=nucltop&blast_rank=9&RID=1Z7910KE01R) |
|  | 10 | [PREDICTED: *Sesamum indicum* transcription factor MYB114-like (LOC105158544), mRNA](http://blast.st-va.ncbi.nlm.nih.gov/Blast.cgi#alnHdr_747054803) | [XM_011075327.1](http://www.ncbi.nlm.nih.gov/nucleotide/747054803?report=genbank&log$=nucltop&blast_rank=10&RID=1Z7910KE01R) |
| MYB114 | 1 | [*Dahlia pinnata* R3myb mRNA for transcription factor R3myb, complete cds](http://blast.st-va.ncbi.nlm.nih.gov/Blast.cgi#alnHdr_327165090) | [AB621921.1](http://www.ncbi.nlm.nih.gov/nucleotide/327165090?report=genbank&log$=nucltop&blast_rank=1&RID=1Z7M31W101R) |
|  | 2 | [*Gynura bicolor* GbMYB2b mRNA for transcription factor MYB2b, complete cds](http://blast.st-va.ncbi.nlm.nih.gov/Blast.cgi#alnHdr_685836931) | [AB581527.1](http://www.ncbi.nlm.nih.gov/nucleotide/685836931?report=genbank&log$=nucltop&blast_rank=2&RID=1Z7M31W101R) |
|  | 3 | [*Gynura bicolor* GbMYB2a mRNA for transcription factor MYB2a, complete cds](http://blast.st-va.ncbi.nlm.nih.gov/Blast.cgi#alnHdr_685836929) | [AB581526.1](http://www.ncbi.nlm.nih.gov/nucleotide/685836929?report=genbank&log$=nucltop&blast_rank=3&RID=1Z7M31W101R) |
|  | 4 | [*Gynura bicolor* GbMYB2 mRNA for transcription factor GbMYB2, complete cds](http://blast.st-va.ncbi.nlm.nih.gov/Blast.cgi#alnHdr_306922321) | [AB550245.1](http://www.ncbi.nlm.nih.gov/nucleotide/306922321?report=genbank&log$=nucltop&blast_rank=4&RID=1Z7M31W101R) |
|  | 5 | [*Gerbera hybrid* cv. 'Terra Regina' mRNA for MYB10 protein](http://blast.st-va.ncbi.nlm.nih.gov/Blast.cgi#alnHdr_40643885) | [AJ554700.1](http://www.ncbi.nlm.nih.gov/nucleotide/40643885?report=genbank&log$=nucltop&blast_rank=5&RID=1Z7M31W101R) |
|  | 6 | [*Gynura bicolor* GbMYB1 mRNA for transcription factor GbMYB1, complete cds](http://blast.st-va.ncbi.nlm.nih.gov/Blast.cgi#alnHdr_306922319) | [AB550244.1](http://www.ncbi.nlm.nih.gov/nucleotide/306922319?report=genbank&log$=nucltop&blast_rank=6&RID=1Z7M31W101R) |
|  | 7 | [*Pericallis cruenta* MYB10 mRNA, partial cds](http://blast.st-va.ncbi.nlm.nih.gov/Blast.cgi#alnHdr_379054200) | [JF499667.1](http://www.ncbi.nlm.nih.gov/nucleotide/379054200?report=genbank&log$=nucltop&blast_rank=7&RID=1Z7M31W101R) |
|  | 8 | [*Chrysanthemum x morifolium* MYB10 mRNA, partial cds](http://blast.st-va.ncbi.nlm.nih.gov/Blast.cgi#alnHdr_379054202) | [JF499668.1](http://www.ncbi.nlm.nih.gov/nucleotide/379054202?report=genbank&log$=nucltop&blast_rank=8&RID=1Z7M31W101R) |
|  | 9 | [*Dahlia pinnata* DvMYB1 mRNA for R2R3-MYB transcriptional factor, complete cds](http://blast.st-va.ncbi.nlm.nih.gov/Blast.cgi#alnHdr_312222648) | [AB601003.1](http://www.ncbi.nlm.nih.gov/nucleotide/312222648?report=genbank&log$=nucltop&blast_rank=9&RID=1Z7M31W101R) |
|  | 10 | [*Actinidia chinensis* MYB family transcription factor mRNA, complete cds](http://blast.st-va.ncbi.nlm.nih.gov/Blast.cgi#alnHdr_543175119) | [KF157390.1](http://www.ncbi.nlm.nih.gov/nucleotide/543175119?report=genbank&log$=nucltop&blast_rank=10&RID=1Z7M31W101R) |
| F3H | 1 | [*Hieracium pilosella* flavanone-3-beta-hydroxylase mRNA, complete cds](http://blast.st-va.ncbi.nlm.nih.gov/Blast.cgi#alnHdr_171906247) | [EU561014.1](http://www.ncbi.nlm.nih.gov/nucleotide/171906247?report=genbank&log$=nucltop&blast_rank=1&RID=22057MHX01R) |
|  | 2 | [*Lactuca sativa* F3H mRNA for flavanone-3-beta-hydroxylase, partial cds](http://blast.st-va.ncbi.nlm.nih.gov/Blast.cgi#alnHdr_299888981) | [AB525910.1](http://www.ncbi.nlm.nih.gov/nucleotide/299888981?report=genbank&log$=nucltop&blast_rank=2&RID=22057MHX01R) |
|  | 3 | [*Dendranthema x grandiflorum* flavanone 3-hydroxylase mRNA, complete cds](http://blast.st-va.ncbi.nlm.nih.gov/Blast.cgi#alnHdr_2801406) | [U86837.1](http://www.ncbi.nlm.nih.gov/nucleotide/2801406?report=genbank&log$=nucltop&blast_rank=3&RID=22057MHX01R) |
|  | 4 | [*Chrysanthemum x morifolium* flavanone 3-hydroxylase mRNA, complete cds](http://blast.st-va.ncbi.nlm.nih.gov/Blast.cgi#alnHdr_347976717) | [JF834892.1](http://www.ncbi.nlm.nih.gov/nucleotide/347976717?report=genbank&log$=nucltop&blast_rank=4&RID=22057MHX01R) |
|  | 5 | [*Nicotiana tabacum* flavanone 3-hydroxylase mRNA, complete cds](http://blast.st-va.ncbi.nlm.nih.gov/Blast.cgi#alnHdr_2828005) | [AF036093.1](http://www.ncbi.nlm.nih.gov/nucleotide/2828005?report=genbank&log$=nucltop&blast_rank=5&RID=22057MHX01R) |
|  | 6 | [*Nicotiana tabacum* flavanone 3-hydroxylase-like mRNA sequence](http://blast.st-va.ncbi.nlm.nih.gov/Blast.cgi#alnHdr_27549590) | [AF036169.1](http://www.ncbi.nlm.nih.gov/nucleotide/27549590?report=genbank&log$=nucltop&blast_rank=6&RID=22057MHX01R) |
|  | 7 | [*Gynura bicolor* GbF3H mRNA for flavanone 3-hydroxylase, complete cds](http://blast.st-va.ncbi.nlm.nih.gov/Blast.cgi#alnHdr_306922333) | [AB550252.1](http://www.ncbi.nlm.nih.gov/nucleotide/306922333?report=genbank&log$=nucltop&blast_rank=7&RID=22057MHX01R) |
|  | 8 | [*Dahlia pinnata* F3H mRNA for flavanone-3-hydroxylase, complete cds](http://blast.st-va.ncbi.nlm.nih.gov/Blast.cgi#alnHdr_308035495) | [AB591828.1](http://www.ncbi.nlm.nih.gov/nucleotide/308035495?report=genbank&log$=nucltop&blast_rank=8&RID=22057MHX01R) |
|  | 9 | [*Pericallis cruenta* flavanone 3-hydroxylase (f3h) mRNA, partial cds](http://blast.st-va.ncbi.nlm.nih.gov/Blast.cgi#alnHdr_94471632) | [DQ471436.1](http://www.ncbi.nlm.nih.gov/nucleotide/94471632?report=genbank&log$=nucltop&blast_rank=9&RID=22057MHX01R) |
|  | 10 | [*Silybum marianum* isolate SmF3'H1 naringenin,2-oxoglutarate 3-dioxygenase-like protein mRNA, partial cds](http://blast.st-va.ncbi.nlm.nih.gov/Blast.cgi#alnHdr_575009756) | [KF767864.1](http://www.ncbi.nlm.nih.gov/nucleotide/575009756?report=genbank&log$=nucltop&blast_rank=10&RID=22057MHX01R) |
| F5H | 1 | [*Chrysanthemum x morifolium* F5H mRNA for ferulate-5-hydroxylase, complete cds](http://blast.st-va.ncbi.nlm.nih.gov/Blast.cgi#alnHdr_418203659) | [AB500858.1](http://www.ncbi.nlm.nih.gov/nucleotide/418203659?report=genbank&log$=nucltop&blast_rank=1&RID=220DZTXK01R) |
|  | 2 | [*Eutrema salsugineum* hypothetical protein (EUTSA_v10024928mg) mRNA, complete cds](http://blast.st-va.ncbi.nlm.nih.gov/Blast.cgi#alnHdr_567216775) | [XM_006411954.1](http://www.ncbi.nlm.nih.gov/nucleotide/567216775?report=genbank&log$=nucltop&blast_rank=2&RID=220DZTXK01R) |
|  | 3 | [*Isatis tinctoria* cytochrome P450 mRNA, complete cds](http://blast.st-va.ncbi.nlm.nih.gov/Blast.cgi#alnHdr_118582213) | [EF103135.1](http://www.ncbi.nlm.nih.gov/nucleotide/118582213?report=genbank&log$=nucltop&blast_rank=3&RID=220DZTXK01R) |
|  | 4 | [*Arabidopsis lyrata* subsp. lyrata ferulate-5-hydroxylase, mRNA](http://blast.st-va.ncbi.nlm.nih.gov/Blast.cgi#alnHdr_297798289) | [XM_002866983.1](http://www.ncbi.nlm.nih.gov/nucleotide/297798289?report=genbank&log$=nucltop&blast_rank=4&RID=220DZTXK01R) |
|  | 5 | [*Arabidopsis lyrata* subsp. petraea partial fah1 gene for ferulate-5-hydroxylase, exons 1-3](http://blast.st-va.ncbi.nlm.nih.gov/Blast.cgi#alnHdr_12578912) | [AJ295586.1](http://www.ncbi.nlm.nih.gov/nucleotide/12578912?report=genbank&log$=nucltop&blast_rank=5&RID=220DZTXK01R) |
|  | 6 | [*Arabidopsis thaliana* fah1 gene for ferulate-5-hydroxylase, exons 1-3 CVI-0 ecotype](http://blast.st-va.ncbi.nlm.nih.gov/Blast.cgi#alnHdr_12578902) | [AJ295581.1](http://www.ncbi.nlm.nih.gov/nucleotide/12578902?report=genbank&log$=nucltop&blast_rank=6&RID=220DZTXK01R) |
|  | 7 | [*Arabidopsis thaliana* fah1 gene for ferulate-5-hydroxylase, exons 1-3 MH-0 ecotype](http://blast.st-va.ncbi.nlm.nih.gov/Blast.cgi#alnHdr_12578900) | [AJ295580.1](http://www.ncbi.nlm.nih.gov/nucleotide/12578900?report=genbank&log$=nucltop&blast_rank=7&RID=220DZTXK01R) |
|  | 8 | [*Arabidopsis thaliana* partial fah1 gene for ferulate-5-hydroxylase, exons 1-2, population variant RV2](http://blast.st-va.ncbi.nlm.nih.gov/Blast.cgi#alnHdr_24740305) | [AJ492859.1](http://www.ncbi.nlm.nih.gov/nucleotide/24740305?report=genbank&log$=nucltop&blast_rank=8&RID=220DZTXK01R) |
|  | 9 | [*Arabidopsis thaliana* partial fah1 gene for ferulate-5-hydroxylase, exons 1-2, population variant TV1](http://blast.st-va.ncbi.nlm.nih.gov/Blast.cgi#alnHdr_24740213) | [AJ492840.1](http://www.ncbi.nlm.nih.gov/nucleotide/24740213?report=genbank&log$=nucltop&blast_rank=9&RID=220DZTXK01R) |
|  | 10 | [*Arabidopsis thaliana* chromosome 4 sequence](http://blast.st-va.ncbi.nlm.nih.gov/Blast.cgi#alnHdr_332656411) | [CP002687.1](http://www.ncbi.nlm.nih.gov/nucleotide/332656411?report=genbank&log$=nucltop&blast_rank=10&RID=220DZTXK01R) |
| CCAoMT | 1 | [*Carthamus tinctorius* CtCoAOMT3 mRNA for caffeoyl CoA O-methyltransferase, complete cds](http://blast.st-va.ncbi.nlm.nih.gov/Blast.cgi#alnHdr_207059701) | [AB430462.1](http://www.ncbi.nlm.nih.gov/nucleotide/207059701?report=genbank&log$=nucltop&blast_rank=1&RID=220KXCD501R) |
|  | 2 | [*Carthamus tinctorius* CtCoAOMT4 mRNA for caffeoyl CoA O-methyltransferase, complete cds](http://blast.st-va.ncbi.nlm.nih.gov/Blast.cgi#alnHdr_207059703) | [AB430463.1](http://www.ncbi.nlm.nih.gov/nucleotide/207059703?report=genbank&log$=nucltop&blast_rank=2&RID=220KXCD501R) |
|  | 3 | [PREDICTED: *Prunus mume* caffeoyl-CoA O-methyltransferase (LOC103335243), mRNA](http://blast.st-va.ncbi.nlm.nih.gov/Blast.cgi#alnHdr_645261817) | [XM_008238253.1](http://www.ncbi.nlm.nih.gov/nucleotide/645261817?report=genbank&log$=nucltop&blast_rank=3&RID=220KXCD501R) |
|  | 4 | [*Prunus persica* hypothetical protein (PRUPE_ppa010497mg) mRNA, complete cds](http://blast.st-va.ncbi.nlm.nih.gov/Blast.cgi#alnHdr_595797107) | [XM_007201175.1](http://www.ncbi.nlm.nih.gov/nucleotide/595797107?report=genbank&log$=nucltop&blast_rank=4&RID=220KXCD501R) |
|  | 5 | [*Eucalyptus camaldulensis* caffeoyl-CoA O-methyltransferase (CCoAOMT) mRNA, complete cds](http://blast.st-va.ncbi.nlm.nih.gov/Blast.cgi#alnHdr_297499578) | [HM106291.1](http://www.ncbi.nlm.nih.gov/nucleotide/297499578?report=genbank&log$=nucltop&blast_rank=5&RID=220KXCD501R) |
|  | 6 | [*Eucalyptus globulus* caffeoyl-CoA 3-O-methyltransferase (CCOMT) mRNA, complete cds](http://blast.st-va.ncbi.nlm.nih.gov/Blast.cgi#alnHdr_3319277) | [AF046122.1](http://www.ncbi.nlm.nih.gov/nucleotide/3319277?report=genbank&log$=nucltop&blast_rank=6&RID=220KXCD501R) |
|  | 7 | [PREDICTED: *Sesamum indicum* caffeoyl-CoA O-methyltransferase (LOC105169526), mRNA](http://blast.st-va.ncbi.nlm.nih.gov/Blast.cgi#alnHdr_747081875) | [XM_011089932.1](http://www.ncbi.nlm.nih.gov/nucleotide/747081875?report=genbank&log$=nucltop&blast_rank=7&RID=220KXCD501R) |
|  | 8 | [*E.gunnii* mRNA for caffeoyl-CoA O-methyltransferase](http://blast.st-va.ncbi.nlm.nih.gov/Blast.cgi#alnHdr_1934858) | [Y12228.1](http://www.ncbi.nlm.nih.gov/nucleotide/1934858?report=genbank&log$=nucltop&blast_rank=8&RID=220KXCD501R) |
|  | 9 | [PREDICTED: *Eucalyptus grandis* caffeoyl-CoA O-methyltransferase (LOC104418719), mRNA](http://blast.st-va.ncbi.nlm.nih.gov/Blast.cgi#alnHdr_702462514) | [XM_010030135.1](http://www.ncbi.nlm.nih.gov/nucleotide/702462514?report=genbank&log$=nucltop&blast_rank=9&RID=220KXCD501R) |
|  | 10 | [PREDICTED: *Nelumbo nucifera* caffeoyl-CoA O-methyltransferase (LOC104601739), mRNA](http://blast.st-va.ncbi.nlm.nih.gov/Blast.cgi#alnHdr_720023950) | [XM_010265186.1](http://www.ncbi.nlm.nih.gov/nucleotide/720023950?report=genbank&log$=nucltop&blast_rank=10&RID=220KXCD501R) |
| GGPS | 1 | [*Lactuca indica* var. laciniata LivlGGPPS mRNA for putative GGPP synthase, partial cds](http://blast.st-va.ncbi.nlm.nih.gov/Blast.cgi#alnHdr_12862618) | [AB055653.1](http://www.ncbi.nlm.nih.gov/nucleotide/12862618?report=genbank&log$=nucltop&blast_rank=1&RID=220TBHCR01R) |
|  | 2 | [*Lactuca indica* var. laciniata LivlGGPPS mRNA for putative GGPP synthase, partial cds](http://blast.st-va.ncbi.nlm.nih.gov/Blast.cgi#alnHdr_12231185) | [AB046111.1](http://www.ncbi.nlm.nih.gov/nucleotide/12231185?report=genbank&log$=nucltop&blast_rank=2&RID=220TBHCR01R) |
|  | 3 | [*Tagetes erecta* GGDP synthase mRNA, complete cds](http://blast.st-va.ncbi.nlm.nih.gov/Blast.cgi#alnHdr_9971807) | [AF251012.1](http://www.ncbi.nlm.nih.gov/nucleotide/9971807?report=genbank&log$=nucltop&blast_rank=3&RID=220TBHCR01R) |
|  | 4 | [*Youngia japonica* YjGGPPs1 mRNA for putative GGPP synthase 1, partial cds](http://blast.st-va.ncbi.nlm.nih.gov/Blast.cgi#alnHdr_13383250) | [AB049083.1](http://www.ncbi.nlm.nih.gov/nucleotide/13383250?report=genbank&log$=nucltop&blast_rank=4&RID=220TBHCR01R) |
|  | 5 | [*Sonchus oleraceus* SoGGPPS mRNA for putative GGPP synthase, partial cds](http://blast.st-va.ncbi.nlm.nih.gov/Blast.cgi#alnHdr_12231183) | [AB046107.1](http://www.ncbi.nlm.nih.gov/nucleotide/12231183?report=genbank&log$=nucltop&blast_rank=5&RID=220TBHCR01R) |
|  | 6 | [*Scoparia dulcis* mRNA for geranylgeranyl pyrophosphate synthase, complete cds](http://blast.st-va.ncbi.nlm.nih.gov/Blast.cgi#alnHdr_6277255) | [AB034250.1](http://www.ncbi.nlm.nih.gov/nucleotide/6277255?report=genbank&log$=nucltop&blast_rank=6&RID=220TBHCR01R) |
|  | 7 | [*Panax notoginseng* isolate CL278Contig6_GGR_reverse geranylgeranyl diphosphate synthase mRNA, complete cds](http://blast.st-va.ncbi.nlm.nih.gov/Blast.cgi#alnHdr_672930594) | [KJ804178.1](http://www.ncbi.nlm.nih.gov/nucleotide/672930594?report=genbank&log$=nucltop&blast_rank=7&RID=220TBHCR01R) |
|  | 8 | [PREDICTED: *Glycine max* geranylgeranyl pyrophosphate synthase, chloroplastic-like (LOC100785539), mRNA](http://blast.st-va.ncbi.nlm.nih.gov/Blast.cgi#alnHdr_571528322) | [XM_003547947.2](http://www.ncbi.nlm.nih.gov/nucleotide/571528322?report=genbank&log$=nucltop&blast_rank=8&RID=220TBHCR01R) |
|  | 9 | [PREDICTED: *Glycine max* geranylgeranyl pyrophosphate synthase, chloroplastic-like (LOC100782362), mRNA](http://blast.st-va.ncbi.nlm.nih.gov/Blast.cgi#alnHdr_571438908) | [XM_003518000.2](http://www.ncbi.nlm.nih.gov/nucleotide/571438908?report=genbank&log$=nucltop&blast_rank=9&RID=220TBHCR01R) |
|  | 10 | [*Glycine max* cDNA, clone: GMFL01-25-G19](http://blast.st-va.ncbi.nlm.nih.gov/Blast.cgi#alnHdr_210141344) | [AK245263.1](http://www.ncbi.nlm.nih.gov/nucleotide/210141344?report=genbank&log$=nucltop&blast_rank=10&RID=220TBHCR01R) |
| ZEP | 1 | [*Lactuca sativa* LsZEP1 mRNA for Lactuca sativa zeaxantin epoxidase 1, complete cds](http://blast.st-va.ncbi.nlm.nih.gov/Blast.cgi#alnHdr_84579403) | [AB120106.1](http://www.ncbi.nlm.nih.gov/nucleotide/84579403?report=genbank&log$=nucltop&blast_rank=1&RID=220ZY6AR01R) |
|  | 2 | [*Chrysanthemum x morifolium* ZEP mRNA for zeaxanthin epoxidase, complete cds](http://blast.st-va.ncbi.nlm.nih.gov/Blast.cgi#alnHdr_87299446) | [AB205053.1](http://www.ncbi.nlm.nih.gov/nucleotide/87299446?report=genbank&log$=nucltop&blast_rank=2&RID=220ZY6AR01R) |
|  | 3 | [*Chrysanthemum boreale* zeaxanthin epoxidase mRNA, complete cds](http://blast.st-va.ncbi.nlm.nih.gov/Blast.cgi#alnHdr_537845529) | [KC202431.1](http://www.ncbi.nlm.nih.gov/nucleotide/537845529?report=genbank&log$=nucltop&blast_rank=3&RID=220ZY6AR01R) |
|  | 4 | [*Camellia sinensis* zeaxanthin epoxidase (zep) mRNA, complete cds](http://blast.st-va.ncbi.nlm.nih.gov/Blast.cgi#alnHdr_743066330) | [KM519985.1](http://www.ncbi.nlm.nih.gov/nucleotide/743066330?report=genbank&log$=nucltop&blast_rank=4&RID=220ZY6AR01R) |
|  | 5 | [PREDICTED: *Nicotiana tomentosiformis* zeaxanthin epoxidase, chloroplastic (LOC104104310), mRNA](http://blast.st-va.ncbi.nlm.nih.gov/Blast.cgi#alnHdr_697113543) | [XM_009612360.1](http://www.ncbi.nlm.nih.gov/nucleotide/697113543?report=genbank&log$=nucltop&blast_rank=5&RID=220ZY6AR01R) |
|  | 6 | [*Daucus carota* subsp. sativus putative zeaxanthin epoxidase mRNA, complete cds](http://blast.st-va.ncbi.nlm.nih.gov/Blast.cgi#alnHdr_79155189) | [DQ192197.1](http://www.ncbi.nlm.nih.gov/nucleotide/79155189?report=genbank&log$=nucltop&blast_rank=6&RID=220ZY6AR01R) |
|  | 7 | [PREDICTED: *Jatropha curcas* zeaxanthin epoxidase, chloroplastic (LOC105639709), mRNA](http://blast.st-va.ncbi.nlm.nih.gov/Blast.cgi#alnHdr_802642417) | [XM_012223843.1](http://www.ncbi.nlm.nih.gov/nucleotide/802642417?report=genbank&log$=nucltop&blast_rank=7&RID=220ZY6AR01R) |
|  | 8 | [PREDICTED: *Populus euphratica* zeaxanthin epoxidase, chloroplastic-like (LOC105138970), mRNA](http://blast.st-va.ncbi.nlm.nih.gov/Blast.cgi#alnHdr_743900477) | [XM_011045237.1](http://www.ncbi.nlm.nih.gov/nucleotide/743900477?report=genbank&log$=nucltop&blast_rank=8&RID=220ZY6AR01R) |
|  | 9 | [PREDICTED: *Malus x domestica* zeaxanthin epoxidase, chloroplastic (LOC103401379), transcript variant X3, mRNA](http://blast.st-va.ncbi.nlm.nih.gov/Blast.cgi#alnHdr_658006323) | [XM_008340095.1](http://www.ncbi.nlm.nih.gov/nucleotide/658006323?report=genbank&log$=nucltop&blast_rank=9&RID=220ZY6AR01R) |
|  | 10 | [PREDICTED: *Malus x domestica* zeaxanthin epoxidase, chloroplastic (LOC103401379), transcript variant X2, mRNA](http://blast.st-va.ncbi.nlm.nih.gov/Blast.cgi#alnHdr_658006321) | [XM_008340094.1](http://www.ncbi.nlm.nih.gov/nucleotide/658006321?report=genbank&log$=nucltop&blast_rank=10&RID=220ZY6AR01R) |
| XET | 1 | [*Dahlia pinnata* xyloglucan endotransglycosylase (XET) mRNA, complete cds](http://blast.st-va.ncbi.nlm.nih.gov/Blast.cgi#alnHdr_300087129) | [HM053613.1](http://www.ncbi.nlm.nih.gov/nucleotide/300087129?report=genbank&log$=nucltop&blast_rank=1&RID=22168N9001R) |
|  | 2 | [*Chrysanthemum x morifolium* xyloglucan endotransglycosylase mRNA, complete cds](http://blast.st-va.ncbi.nlm.nih.gov/Blast.cgi#alnHdr_302035347) | [HM752243.1](http://www.ncbi.nlm.nih.gov/nucleotide/302035347?report=genbank&log$=nucltop&blast_rank=2&RID=22168N9001R) |
|  | 3 | [*Tagetes patula* xyloglucan endotransglucosylase/hydrolase (XTH) mRNA, complete cds](http://blast.st-va.ncbi.nlm.nih.gov/Blast.cgi#alnHdr_340396651) | [JN164663.1](http://www.ncbi.nlm.nih.gov/nucleotide/340396651?report=genbank&log$=nucltop&blast_rank=3&RID=22168N9001R) |
|  | 4 | [*Gossypium hirsutum* cultivar Coker 312 xyloglucan endotransglucosylase/hydrolase (XTH2) mRNA, complete cds](http://blast.st-va.ncbi.nlm.nih.gov/Blast.cgi#alnHdr_308229783) | [HM749061.1](http://www.ncbi.nlm.nih.gov/nucleotide/308229783?report=genbank&log$=nucltop&blast_rank=4&RID=22168N9001R) |
|  | 5 | [PREDICTED: *Nicotiana sylvestris* probable xyloglucan endotransglucosylase/hydrolase protein 6 (LOC104223372), mRNA](http://blast.st-va.ncbi.nlm.nih.gov/Blast.cgi#alnHdr_698564887) | [XM_009774799.1](http://www.ncbi.nlm.nih.gov/nucleotide/698564887?report=genbank&log$=nucltop&blast_rank=5&RID=22168N9001R) |
|  | 6 | [PREDICTED: *Nicotiana tomentosiformis* probable xyloglucan endotransglucosylase/hydrolase protein 7 (LOC104118549), mR](http://blast.st-va.ncbi.nlm.nih.gov/Blast.cgi#alnHdr_697147884)NA | [XM_009629819.1](http://www.ncbi.nlm.nih.gov/nucleotide/697147884?report=genbank&log$=nucltop&blast_rank=6&RID=22168N9001R) |
|  | 7 | [PREDICTED: *Nicotiana sylvestris* probable xyloglucan endotransglucosylase/hydrolase protein 7 (LOC104239360), mRNA](http://blast.st-va.ncbi.nlm.nih.gov/Blast.cgi#alnHdr_698491768) | [XM_009793982.1](http://www.ncbi.nlm.nih.gov/nucleotide/698491768?report=genbank&log$=nucltop&blast_rank=7&RID=22168N9001R) |
|  | 8 | [PREDICTED: *Nicotiana tomentosiformis* probable xyloglucan endotransglucosylase/hydrolase protein 7 (LOC104105066), mRNA](http://blast.st-va.ncbi.nlm.nih.gov/Blast.cgi#alnHdr_697115349) | [XM_009613303.1](http://www.ncbi.nlm.nih.gov/nucleotide/697115349?report=genbank&log$=nucltop&blast_rank=8&RID=22168N9001R) |
|  | 9 | [*Nicotiana tabacum* xyloglucan endotransglucosylase-hydrolase XTH7 (XTH7) mRNA, complete cds](http://blast.st-va.ncbi.nlm.nih.gov/Blast.cgi#alnHdr_662552202) | [KJ730270.1](http://www.ncbi.nlm.nih.gov/nucleotide/662552202?report=genbank&log$=nucltop&blast_rank=9&RID=22168N9001R) |
|  | 10 | [*Lotus japonicus* clone JCVI-FLLj-3M17 unknown mRNA](http://blast.st-va.ncbi.nlm.nih.gov/Blast.cgi#alnHdr_388496159) | [BT136351.1](http://www.ncbi.nlm.nih.gov/nucleotide/388496159?report=genbank&log$=nucltop&blast_rank=10&RID=22168N9001R) |
| MYB44 | 1 | [PREDICTED: *Solanum lycopersicum* transcription factor MYB44-like (LOC101244577), mRNA](http://blast.st-va.ncbi.nlm.nih.gov/Blast.cgi#alnHdr_723695239) | [XM_004238075.2](http://www.ncbi.nlm.nih.gov/nucleotide/723695239?report=genbank&log$=nucltop&blast_rank=1&RID=221B4KJM01R) |
|  | 2 | [*Solanum lycopersicum* chromosome ch04, complete genome](http://blast.st-va.ncbi.nlm.nih.gov/Blast.cgi#alnHdr_663680879) | [HG975516.1](http://www.ncbi.nlm.nih.gov/nucleotide/663680879?report=genbank&log$=nucltop&blast_rank=2&RID=221B4KJM01R) |
|  | 3 | [PREDICTED: *Solanum tuberosum* transcription factor MYB44-like (LOC102581745), mRNA](http://blast.st-va.ncbi.nlm.nih.gov/Blast.cgi#alnHdr_565403963) | [XM_006367359.1](http://www.ncbi.nlm.nih.gov/nucleotide/565403963?report=genbank&log$=nucltop&blast_rank=3&RID=221B4KJM01R) |
|  | 4 | [*Solanum lycopersicum* cDNA, clone: LEFL3156L15, HTC in root](http://blast.st-va.ncbi.nlm.nih.gov/Blast.cgi#alnHdr_225319406) | [AK329785.1](http://www.ncbi.nlm.nih.gov/nucleotide/225319406?report=genbank&log$=nucltop&blast_rank=4&RID=221B4KJM01R) |
|  | 5 | [*Solanum lycopersicum* cDNA, clone: LEFL2016K12, HTC in fruit](http://blast.st-va.ncbi.nlm.nih.gov/Blast.cgi#alnHdr_225312161) | [AK326899.1](http://www.ncbi.nlm.nih.gov/nucleotide/225312161?report=genbank&log$=nucltop&blast_rank=5&RID=221B4KJM01R) |
|  | 6 | [*Solanum tuberosum* clone 16 tuber-specific and sucrose-responsive element binding factor (TSF) mRNA, partial cds](http://blast.st-va.ncbi.nlm.nih.gov/Blast.cgi#alnHdr_9954113) | [AF122052.1](http://www.ncbi.nlm.nih.gov/nucleotide/9954113?report=genbank&log$=nucltop&blast_rank=6&RID=221B4KJM01R) |
|  | 7 | [*Solanum lycopersicum* cDNA, clone: LEFL1098BH02, HTC in leaf](http://blast.st-va.ncbi.nlm.nih.gov/Blast.cgi#alnHdr_225321571) | [AK325588.1](http://www.ncbi.nlm.nih.gov/nucleotide/225321571?report=genbank&log$=nucltop&blast_rank=7&RID=221B4KJM01R) |
|  | 8 | [*Solanum pennellii* chromosome ch04, complete genome](http://blast.st-va.ncbi.nlm.nih.gov/Blast.cgi#alnHdr_663673442) | [HG975443.1](http://www.ncbi.nlm.nih.gov/nucleotide/663673442?report=genbank&log$=nucltop&blast_rank=8&RID=221B4KJM01R) |
|  | 9 | [*A.thaliana* mRNA for MYB-related protein (1195 bp)](http://blast.st-va.ncbi.nlm.nih.gov/Blast.cgi#alnHdr_1263094) | [Z54136.1](http://www.ncbi.nlm.nih.gov/nucleotide/1263094?report=genbank&log$=nucltop&blast_rank=9&RID=221B4KJM01R) |
|  | 10 | [Cloning vector pTACAtg1 DNA, complete sequence](http://blast.st-va.ncbi.nlm.nih.gov/Blast.cgi#alnHdr_665899259) | [AB904499.1](http://www.ncbi.nlm.nih.gov/nucleotide/665899259?report=genbank&log$=nucltop&blast_rank=10&RID=221B4KJM01R) |
| APX | 1 | [PREDICTED: *Nicotiana sylvestris* L-ascorbate peroxidase 3, peroxisomal-like (LOC104243325), mRNA](http://blast.st-va.ncbi.nlm.nih.gov/Blast.cgi#alnHdr_698502290) | [XM_009798505.1](http://www.ncbi.nlm.nih.gov/nucleotide/698502290?report=genbank&log$=nucltop&blast_rank=1&RID=221GNWZ301R) |
|  | 2 | [PREDICTED: *Jatropha curcas* L-ascorbate peroxidase 3, peroxisomal-like (LOC105647126), mRNA](http://blast.st-va.ncbi.nlm.nih.gov/Blast.cgi#alnHdr_802753529) | [XM_012233123.1](http://www.ncbi.nlm.nih.gov/nucleotide/802753529?report=genbank&log$=nucltop&blast_rank=2&RID=221GNWZ301R) |
|  | 3 | [*Morus notabilis* L-ascorbate peroxidase 3 partial mRNA](http://blast.st-va.ncbi.nlm.nih.gov/Blast.cgi#alnHdr_703163385) | [XM_010115021.1](http://www.ncbi.nlm.nih.gov/nucleotide/703163385?report=genbank&log$=nucltop&blast_rank=3&RID=221GNWZ301R) |
|  | 4 | [*Oryza sativa* Japonica Group clone KCS004G09 L-ascorbate peroxidase mRNA, complete cds](http://blast.st-va.ncbi.nlm.nih.gov/Blast.cgi#alnHdr_341870576) | [HQ013288.1](http://www.ncbi.nlm.nih.gov/nucleotide/341870576?report=genbank&log$=nucltop&blast_rank=4&RID=221GNWZ301R) |
|  | 5 | [*Oryza rufipogon* (W1943) cDNA clone: ORW1943C004J17, full insert sequence](http://blast.st-va.ncbi.nlm.nih.gov/Blast.cgi#alnHdr_150171366) | [CU405801.1](http://www.ncbi.nlm.nih.gov/nucleotide/150171366?report=genbank&log$=nucltop&blast_rank=5&RID=221GNWZ301R) |
|  | 6 | [*Oryza sativa* (indica cultivar-group) cDNA clone:OSIGCSN015H07, full insert sequence](http://blast.st-va.ncbi.nlm.nih.gov/Blast.cgi#alnHdr_116632634) | [CT832438.1](http://www.ncbi.nlm.nih.gov/nucleotide/116632634?report=genbank&log$=nucltop&blast_rank=6&RID=221GNWZ301R) |
|  | 7 | [*Oryza sativa* (indica cultivar-group) cDNA clone:OSIGCRA102O15, full insert sequence](http://blast.st-va.ncbi.nlm.nih.gov/Blast.cgi#alnHdr_116632632) | [CT832436.1](http://www.ncbi.nlm.nih.gov/nucleotide/116632632?report=genbank&log$=nucltop&blast_rank=7&RID=221GNWZ301R) |
|  | 8 | [*Oryza sativa* Japonica Group Os08g0549100 (Os08g0549100) mRNA, complete cds](http://blast.st-va.ncbi.nlm.nih.gov/Blast.cgi#alnHdr_115477686) | [NM_001068974.1](http://www.ncbi.nlm.nih.gov/nucleotide/115477686?report=genbank&log$=nucltop&blast_rank=8&RID=221GNWZ301R) |
|  | 9 | [*Oryza sativa* Japonica Group cDNA clone:J023074O14, full insert sequence](http://blast.st-va.ncbi.nlm.nih.gov/Blast.cgi#alnHdr_32980865) | [AK070842.1](http://www.ncbi.nlm.nih.gov/nucleotide/32980865?report=genbank&log$=nucltop&blast_rank=9&RID=221GNWZ301R) |
|  | 10 | [*Oryza rufipogon* (W1943) cDNA clone: ORW1943C103I13, full insert sequence](http://blast.st-va.ncbi.nlm.nih.gov/Blast.cgi#alnHdr_157887856) | [CT841589.1](http://www.ncbi.nlm.nih.gov/nucleotide/157887856?report=genbank&log$=nucltop&blast_rank=10&RID=221GNWZ301R) |
